# METTL3 regulates thyroid cancer differentiation and chemosensitivity by modulating PAX8

**DOI:** 10.7150/ijbs.84797

**Published:** 2024-06-17

**Authors:** Ning Kang, Zewei Zhao, Zhongyu Wang, Junya Ning, Huijuan Wang, Wei Zhang, Xianhui Ruan, Ming Gao, Xiangqian Zheng

**Affiliations:** 1 Department of Thyroid and Neck Cancer, Tianjin Medical University Cancer Institute & Hospital, National Clinical Research Center for Cancer, Key Laboratory of Cancer Prevention and Therapy, Tianjin's Clinical Research Center for Cancer, Tianjin 300060, China.; 2 Department of Breast and Thyroid Surgery, Tianjin Union Medical Center, No. 190 Jieyuan Road, Hongqiao District, Tianjin 300121, China.

**Keywords:** Thyroid cancer dedifferentiation, chemotherapeutic resistance, N6-methyladenosine (m^6^A) modification, METTL3, PAX8.

## Abstract

**Background:** Thyroid cancer (TC) is a common endocrine cancer with a favourable prognosis. However, poor patient prognosis due to TC dedifferentiation is becoming an urgent challenge. Recently, methyltransferase-like 3 (METTL3)-mediated *N^6^*-methyladenosine (m^6^A) modification has been demonstrated to play an important role in the occurrence and progression of various cancers and a tumour suppressor role in TC. However, the mechanism of METTL3 in TC remains unclear.

**Methods:** The correlation between METTL3 and prognosis in TC patients was evaluated by immunohistochemistry. *Mettl3^fl/fl^Braf^V600E^TPO-cre* TC mouse models and RNA-seq were used to investigate the underlying molecular mechanism, which was further validated by *in vitro* experiments. The target gene of METTL3 was identified, and the complete m^6^A modification process was described. The phenomenon of low expression of METTL3 in TC was explained by identifying miRNAs that regulate METTL3.

**Results:** We observed that METTL3 expression was negatively associated with tumour progression and poor prognosis in TC. Mechanistically, silencing METTL3 promoted the progression and dedifferentiation of papillary thyroid carcinoma (PTC) both *in vivo* and *in vitro*. Moreover, overexpressing METTL3 promoted the sensitivity of PTC and anaplastic thyroid cancer (ATC) cells to chemotherapeutic drugs and iodine-131 (^131^I) administration. Overall, the METTL3/PAX8/YTHDC1 axis has been revealed to play a pivotal role in repressing tumour occurrence, and is antagonized by miR-493-5p.

## Introduction

The incidence rate of TC continues to increase worldwide, and it is projected that more than 3.7 million new cases will be diagnosed in the 2028-2032 period in mainland China^1^. Research on TC pathogenesis and improvement of its treatment has had a high profile in recent years. Differentiated TC (DTC) is the most common pathological type of thyroid tumour and has a favourable prognosis with standard treatments, such as surgery, administration of radioactive iodine (RAI) and thyroid-stimulating hormone (TSH) suppressive therapy. However, among DTC patients, approximately 7% develop metastatic disease. Fifty to seventy percent of these patients exhibit loss of iodine-131 (^131^I) uptake initially or gradually, also known as RAI-refractory DTC (RAIR-DTC) and their 10-year survival rate is then reduced to less than 20%^2^. Among these patients, dedifferentiation is the main reason that leads to PTC transform into poorly differentiated TC (PDTC) or anaplastic TC (ATC). Although there are many chemotherapeutic drugs in clinical use such as Paclitaxel, Doxorubicin, Cisplatin, Docetaxel, Carboplatin, Dabrafenib and Trametinib, the prognosis for ATC patients is poor because chemo-resistance is common.

The molecular mechanisms underlying the dedifferentiation and chemoresistance in PDTC and ATC deserve in-depth investigation to provide more effective therapeutic strategies. It has been reported that CRSP8 could suppress TC differentiation and regulate the sensitivity of TC cells to chemotherapeutics via targeting IKKα signaling^3^. TRIM11, which acts as a post-translational modulating factor of Hippo pathway influence cell sensitivity to chemotherapy^4^. Such therapeutics based on the mutational of tumors might be helpful in overcoming therapeutic resistance in PDTC and ATC.

According to existing research, genetic alterations are the fundamental drivers for the tumorigenesis and pathogenesis of TC. Aberrant activation of the mitogen-activated protein kinase (MAPK) and phosphoinositide 3-kinase (PI3K) pathways occurs, followed by dysfunction of the Na^+^/I^-^ symporter (NIS), which can determine the iodine uptake capacity of thyroid follicular cells^5^, ^6^. Paired box 8 (PAX8), as a member of the pair box gene (PAX) family and an important component of the cytoskeleton, can govern the transcription of NIS and is necessary for the development of the thyroid gland and for maintaining the differentiated state in the mature thyroid^7^.

m^6^A methylation has been shown to be one of the most important epigenetic regulatory mechanisms in the occurrence and development of various cancers. Moreover, many m^6^A-derived diagnostic and prognostic biomarkers have been identified in the past few years. The mRNA-seq and MeRIP-qPCR results from our previous study showed that ACSM5, which is downregulated in TC and inversely correlated with papillary thyroid carcinoma (PTC) malignancy and patient survival, could be regulated by m^6^A methylation of mRNA^8^. METTL3, as the writer of m^6^A, plays a pivotal tumour-suppressor role in PTC carcinogenesis through c-Rel and RelA inactivation of the nuclear factor κB (NF-κB) pathway to regulate tumour growth^9^.

Genetically engineered *Braf^V600E^* mouse models have been developed to provide a more realistic *in vivo* research environment^10^, ^11^. Thyroid expression of *Braf^V600E^* leads to the malignant transformation of thyroid epithelial cells to PTC cells, which rapidly progresses to DTC in mice.

In the present study, we investigated the antitumour activity of METTL3 in *Braf^V600E^* knock-in and *Mettl3* knockout mice. We found that silencing METTL3 promoted the dedifferentiation of TC and shortened the survival period of mice significantly. High METTL3 not only inhibited the malignant biological behavior like proliferation and migration of TC cells, but also increased sensibility toward chemotherapeutics. We explored the association of METTL3 with TC differentiation and reported a potential treatment for thyroid cancer.

## Results

### Compared with normal thyroid tissues, the expression level of METTL3 in TC tissues is low

**Figure 1:**
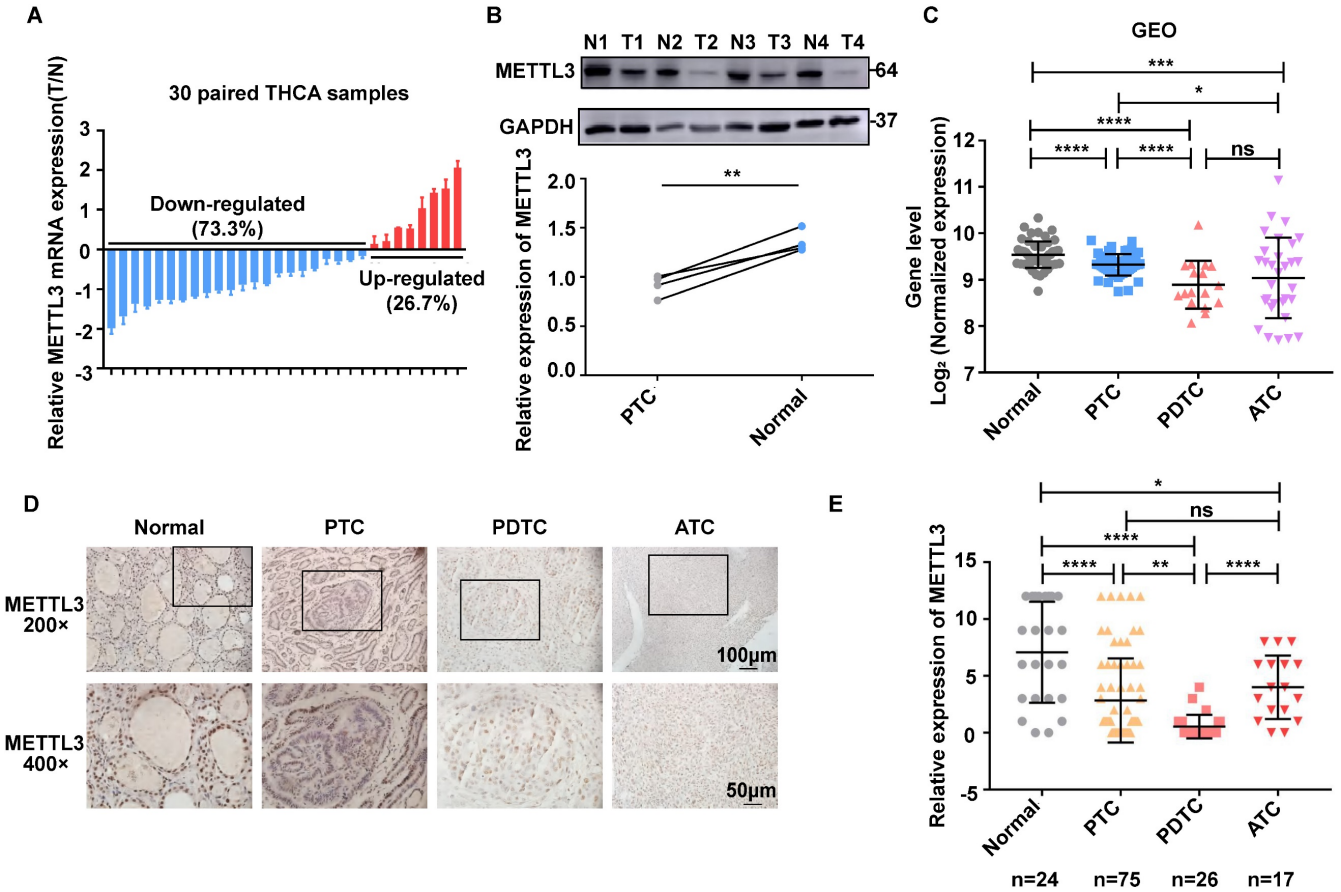
** METTL3 expression is downregulated in human TC. (A)** Quantitative real-time PCR was used to measure the mRNA level of METTL3 in normal and thyroid cancer tissues. **(B)** The protein expression levels of METTL3 were examined by western blotting in normal and thyroid cancer tissues. **(C)** The expression of METTL3 in human normal thyroid cells, PTC cells, PDTC cells and ATC cells was mined from the GEO datasets. **(D)** Representative IHC analysis of METTL3 expression in different TC patient specimens. The scale bars are 50 μm and 100 μm. **(E)** IHC staining score statistics to measure the mRNA level of METTL3 in normal thyroid tissues, PTC tissues, PDTC tissues and ATC tissues. Data represents mean ± SEM, *p< 0.05, **p < 0.01, ***p < 0.001, ****p < 0.0001.

To evaluate the role of METTL3 in TC progression, we first conducted RT‒qPCR analysis and observed that METTL3 displayed higher mRNA levels in normal thyroid tissues than in PTC tissues (Fig. 1A). Furthermore, the protein levels of METTL3 were decreased in PTC tissues compared with those of adjacent tissues (Fig. 1B). In addition, the expression of METTL3 in human normal thyroid cells, PTC cells, PDTC cells and ATC cells was evaluated using GEO datasets (GSE3467^12^, GSE33630^13^, ^14^, and GSE76039^15^). The results showed that METTL3 expression in thyroid carcinoma was significantly lower than that in normal thyroid tissue and was positively correlated with the differentiation level of thyroid carcinoma (Fig. 1C). Consistent with this finding, we measured METTL3 protein levels with IHC and found that METTL3 staining was mainly concentrated in the nucleus, and its expression level in TC tissue was significantly reduced compared with that in normal tissue (Fig. 1D-E). Moreover, chi-square analysis further suggested that low METTL3 expression was positively correlated with advanced T stage, lymph node metastasis and the American Joint Committee on Cancer (AJCC)-defined stage of extrathyroidal extension (Supplementary Table 1). Together, these findings suggest that METTL3 is expressed at low levels in TC and related to the differentiation status of TC.

### Knockout METTL3 in *Braf^V600E^TPO-cre* mice significantly promoted dedifferentiation of TC cells and tumor progression

**Figure 2:**
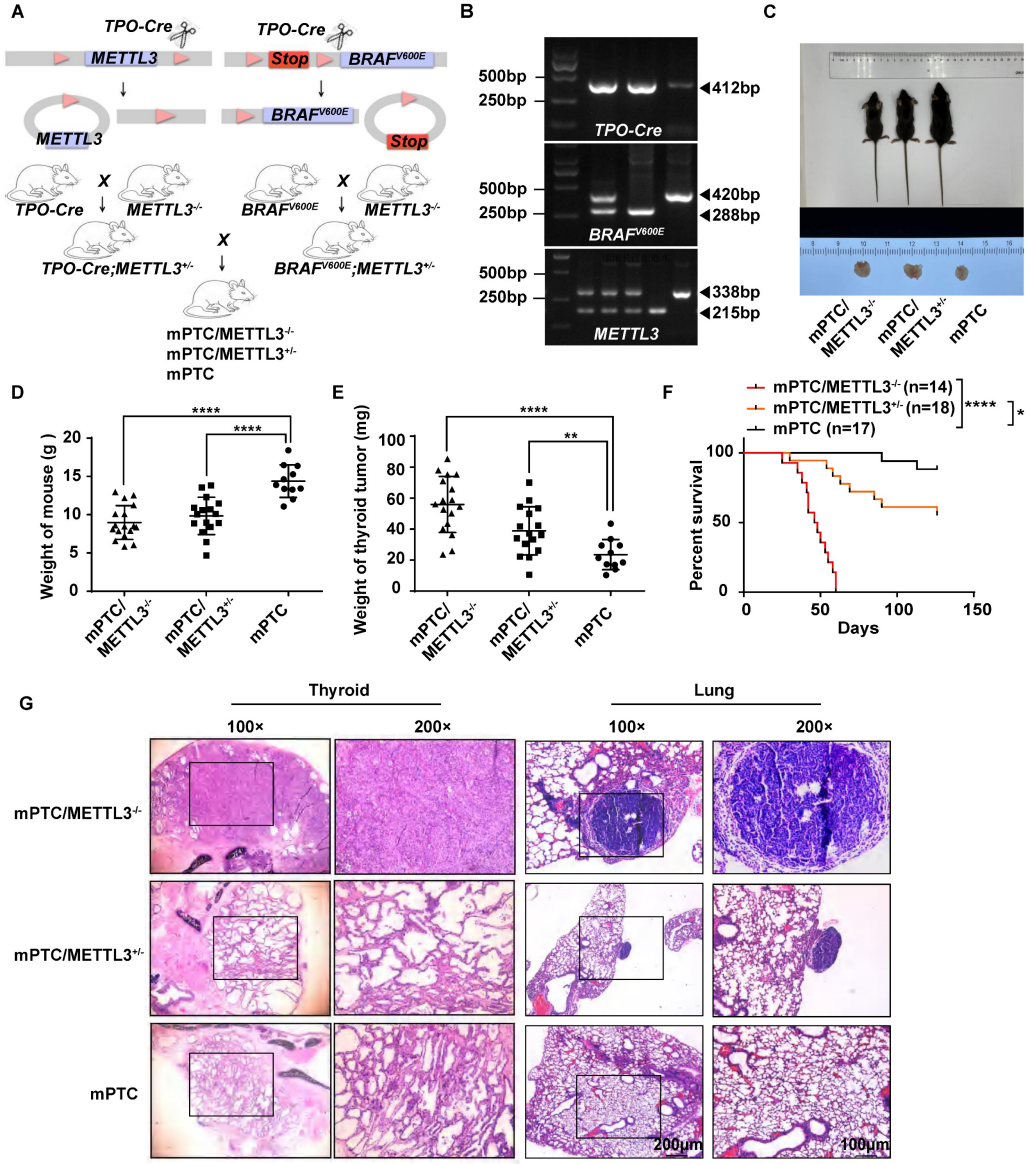
** Generation of *Mettl3^fl/fl^Braf^V600E^TPO-cre* TC conditional mouse models. (A)** Schematic of the construction of spontaneous TC tumorigenesis mouse models. **(B)** The genotypes of mice were identified, and DNA gel imaging showed that the band sizes of *TPO-cre, BRAF^V600E^* and *METTL3**^fl/fl^*** were 412 bp, 420 bp/288 bp, and 338 bp/215 bp, respectively. **(C)** This image represents the entire thyroid tissue and corresponding body size of a group of mPTC, mPTC/METTL3^+/-^, and mPTC/METTL3^-/-^ mice at 5 weeks.** (D)** Body weight of mPTC, mPTC/METTL3^+/-^ and mPTC/METTL3^-/-^ mice at 5 weeks, n=11 mPTC, n=16 mPTC/METTL3^+/-^, n=17 mPTC/METTL3^-/-^. **(E)** Weight of TC tissue from mPTC, mPTC/METTL3^+/-^ and mPTC/MET TL3^-/-^ mice at 5 weeks, n=11 mPTC, n=16 mPTC/METTL3^+/-^, n=17 mPTC/METT3^-/-^. **(F)** Survival curve of mice bearing mPTC (n=17), mPTC/METTL3^+/-^ (n=18) and mPTC/METTL3^-/-^ (n =14) tumours. **(G)** Representative H&E staining of thyroid tissues from mPTC, mPTC/METTL3^+/-^ and mPTC/METTL3^-/-^ mice. The scale bars are 100 μm and 200 μm. Data represents mean ± SEM, *p< 0.05, **p < 0.01, ***p < 0.001, ****p < 0.0001.

To investigate the physiological roles of METTL3 in the pathogenesis of TC *in vivo*, we constructed a mouse model of spontaneous tumorigenic TC coupled with *Mettl3* knockout by hybridizing *Mettl3^fl/fl^* with *TPO-cre* and *Braf^V600E^* mice. The offspring of mice were crossed to obtain triple transgenic mice (Fig. 2A-B). Once the mice reached 5 weeks old, they were weighed and sacrificed for thyroid tissue collection. Surprisingly, compared with counterpart mice, *Mettl3* KO mice grew significantly slower and were smaller in size. In addition, thyroid tumours were larger in *Mettl3* KO mice than in WT mice (Fig. 2C). The weights of *Mettl3* KO and heterozygous (HET) mice were significantly lower than those of WT mice (Fig. 2D). The thyroid tumours in the KO and HET groups were weighted more than those in the control group (Fig. 2E). As a result, survival in the KO group was significantly shortened (Fig. 2F). Interestingly, the thyroid tissue derived from *Mettl3* KO mice showed normal thyroid follicle loss instead of exhibiting closely arranged cancer cells, even in 5-week-old mice. The HET group mice displayed a similar phenotype to the control group, where the thyroid follicles were disordered similar to LSL-*Braf^V600E^*/*TPO-cre* model mice^16^. In addition, compared with HET metastatic tumours, *Mettl3* KO also promoted pulmonary tumour metastasis with a papillary structure (Fig. 2G).

### The overexpression of METTL3 enhanced the chemosensitivity of TC cells

**Figure 3:**
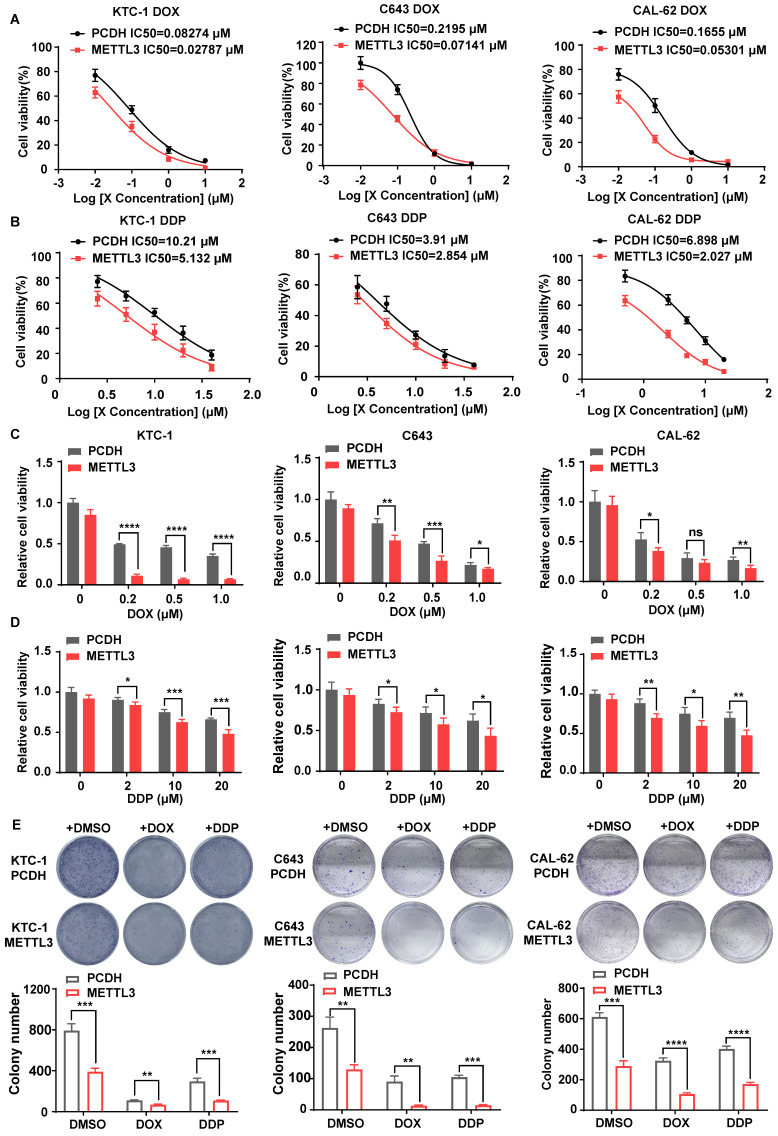
** METTL3 overexpression enhanced the chemosensitivity of TC cells. (A)** Doxorubicin IC_50_ detection by a CCK-8 assay in PCDH-transfected C643, CAL-62, and KTC-1 cells and OE METTL3-transfected C643, CAL-62, and KTC-1 cells. **(B)** Cisplatin IC_50_ detection by a CCK-8 assay in PCDH-transfected C643, CAL-62, and KTC-1 cells and OE METTL3-transfected C643, CAL-62, and KTC-1 cells. **(C)** Cell viability of OE METTL3-transfected C643, CAL-62, KTC-1 cells and control cells after treatment with doxorubicin in different concentration gradients for 48 h. **(D)** Cell viability of OE METTL3-transfected C643, CAL-62, KTC-1 cells and control cells after treatment with cisplatin in different concentration gradients for 48 h. **(E)** METTL3-overexpressing C643, CAL-62, and KTC-1 cells and control cells were maintained in medium containing doxorubicin or cisplatin for 48 hours, followed by colony formation assays. Data represents mean ± SEM, *p< 0.05, **p < 0.01, ***p < 0.001, ****p < 0.0001.

Considering the low expression of METTL3 in PDTC and ATC and its depletion leading to the loss of normal thyroid tissue structure in mouse models, we speculated that METTL3 may affect thyroid differentiation, a process that largely contributes to overcoming tumour chemoresistance^17^, ^18^. Thus, we hypothesized that METTL3 might affect TC sensitivity to chemotherapeutics, such as doxorubicin (DOX) and cisplatin (DDP). First, we measured the expression level of METTL3 protein in thyroid follicular epithelial cells, PTC cells and ATC cells using western blot analysis. The expression of METTL3 in K-1 and KTC-1 (PTC) cells and C643 and CAL-62 (ATC) cells was significantly downregulated than that in other cells (Supplementary Fig. 1A). Accordingly, we engineered cell lines with stable overexpression of METTL3 in KTC-1, C643 and CAL-62 cells (Supplementary Fig. 1B), from which m^6^A modification levels were markedly increased (Supplementary Fig. 1C). Consistent with previous findings^9^, overexpression of METTL3 inhibited TC cell migration, proliferation and cell viability (Supplementary Fig. 1D-F). Moreover, METTL3 overexpression also increased the sensitivity of TC cell lines CAL-62, C643 and KTC-1 to DDP (IC50=2.027, IC50=2.854, IC50=5.132) and DOX (IC50=0.05301, IC50=0.07141, IC50=0.02787) compared with that of the control cells (Fig. 3A-B). These findings were further confirmed with CCK8 (Fig. 3C-D) and colony formation assays (Fig. 3E), suggesting that METTL3 expression could sensitize TC cells to DDP and DOX treatment. Since ^131^I is commonly employed for certain TC therapies, we explored the potential function of METTL3 in affecting ^131^I-mediated TC therapy. The iodine uptake capacity of TC cells was measured, and the results showed that overexpression of METTL3 strongly improved TC cells uptake of ^131^I (Supplementary Fig. 1G), indicating an adjuvant effect of METTL3 for ^131^I-mediated TC therapy.

### METTL3 deletion promotes TC dedifferentiation

**Figure 4:**
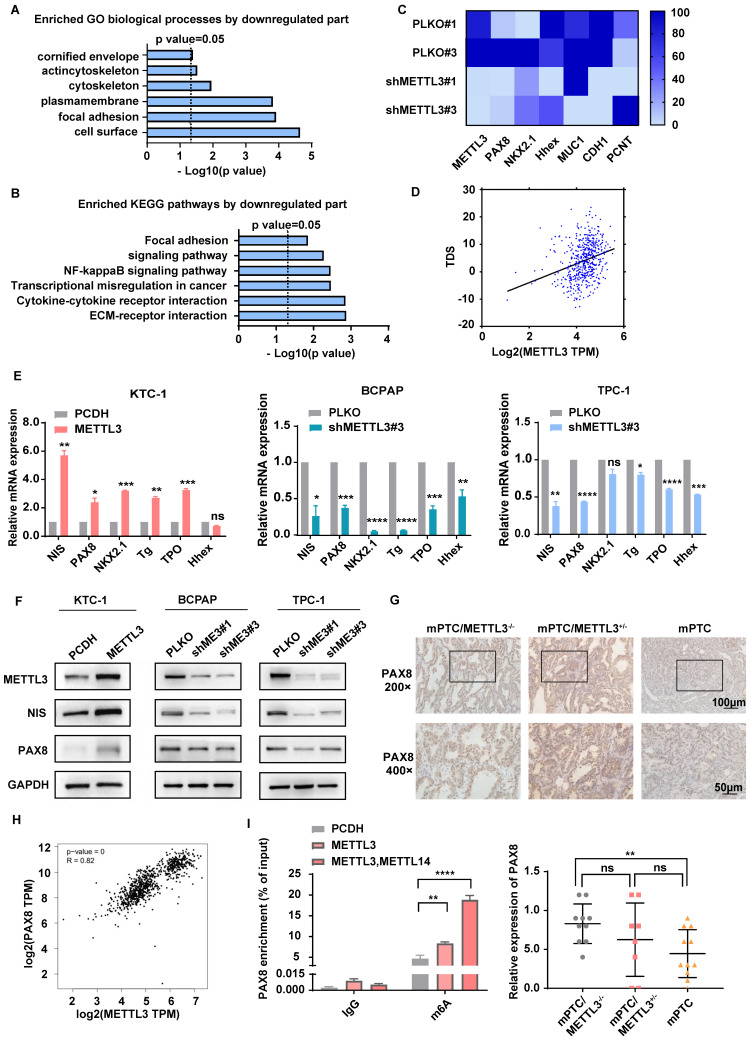
** METTL3 deletion promotes TC dedifferentiation. (A&B)** Representative GO term analysis and KEGG pathway analysis of upregulated and downregulated genes after transfection with shMETTL3. **(C)** Representative gene expression in BCPAP cells after transfection with shMETTL3. **(D)** Correlation between METTL3 expression and the TDS score. **(E)** The RNA expression levels of thyroid differentiation-related genes were examined by quantitative real-time PCR in OE METTL3-transfected KTC-1 cells, shMETTL3-transfected BCPAP cells and TPC-1 cells. **(F)** The protein expression levels of NIS and PAX8 were examined by western blotting in OE METTL3-transfected KTC-1 cells, shMETTL3-transfected TPC-1 cells and BCPAP cells. **(G)** IHC staining of PAX8 in mPTC, mPTC/METTL3^+/-^ and mPTC/METTL3^-/-^. The scale bars are 100 μm and 50 μm. **(H)** Bioinformatics analysis using data from TCGA database showed that PAX8 expression was positively correlated with METTL3 levels. **(I)** MeRIP assay results showed increased m^6^A modification levels of PAX8 in KTC-1 cells that overexpressed METTL3 or METTL14. Data represents mean ± SEM, *p< 0.05, **p < 0.01, ***p < 0.001, ****p < 0.0001.

To further investigate the molecular mechanism of METTL3 in repressing TC, we conducted transcriptomic analyses with RNA-Seq in *METTL3*-depleted cells (Supplementary Fig. 2A). Dot blot analysis proved that the downregulation of METTL3 in TC cells indeed reduced the level of m^6^A modification (Supplementary Fig. 2B). According to Gene Ontology (GO) results, the differently expressed genes derived from *METTL3*-depleted cells were enriched in cellular components such as the cell surface and plasma membrane and were functionally associated with focal adhesion (Fig. 4A). Interestingly, Kyoto Encyclopedia of Genes and Genomes (KEGG) analyses identified that the ECM-receptor interaction signalling pathway, a pathway tightly associated with intercellular communication, cell proliferation, adhesion and migration^19^, ^20^, was significantly increased upon *METTL3* knockdown (Fig. 4B). Previous studies also demonstrated that the convergence of ECM and thyroid hormone on integrin αvβ3 is crucial to the proliferation and self-renewal of cortical progenitor cells^21^. Moreover, we proved that the low expression of METTL3 promotes migration ability, cell proliferation, and cell viability through transwell experiments, cell cloning experiments and CCK8 assays (Supplementary Fig. 2C-E).

Loss of thyroid differentiation marker expression is one of the hallmarks of advanced TCs. Iodine therapy is ineffective for some patients due to the reduction of NIS levels. According to the results of RNA-Seq, *METTL3* knockdown was accompanied by decreased thyroid differentiation-related genes, such as PAX8, NKX2.1, and Hhex (Fig. 4C). Moreover, the connection of METTL3 with the thyroid differentiation score (TDS), which contains 16 genes related to iodine metabolism and thyroid regulation, was analysed^15^. Surprisingly, METTL3 expression was positively correlated with the TDS score (Fig. 4D). Quantitative analysis demonstrated that NIS, PAX8, NKX2.1, TG, TPO and Hhex levels were significantly decreased upon METTL3 deletion (Fig. 4E), which suggests that METTL3 promotes TC dedifferentiation and performs a tumour suppressor role. PAX8 plays pivotal roles in the development of thyroid follicular cells by promoting the expression of thyroid-specific genes^22^. Here, we observed that both the mRNA and protein levels of PAX8 and NIS were elevated upon expression of METTL3 (Fig. 4F), which was further validated in *Mettl3*-KO mouse TC tissues (Fig. 4G).

### METTL3 regulates the expression of PAX8 through m^6^A modification in thyroid carcinoma

**Figure 5:**
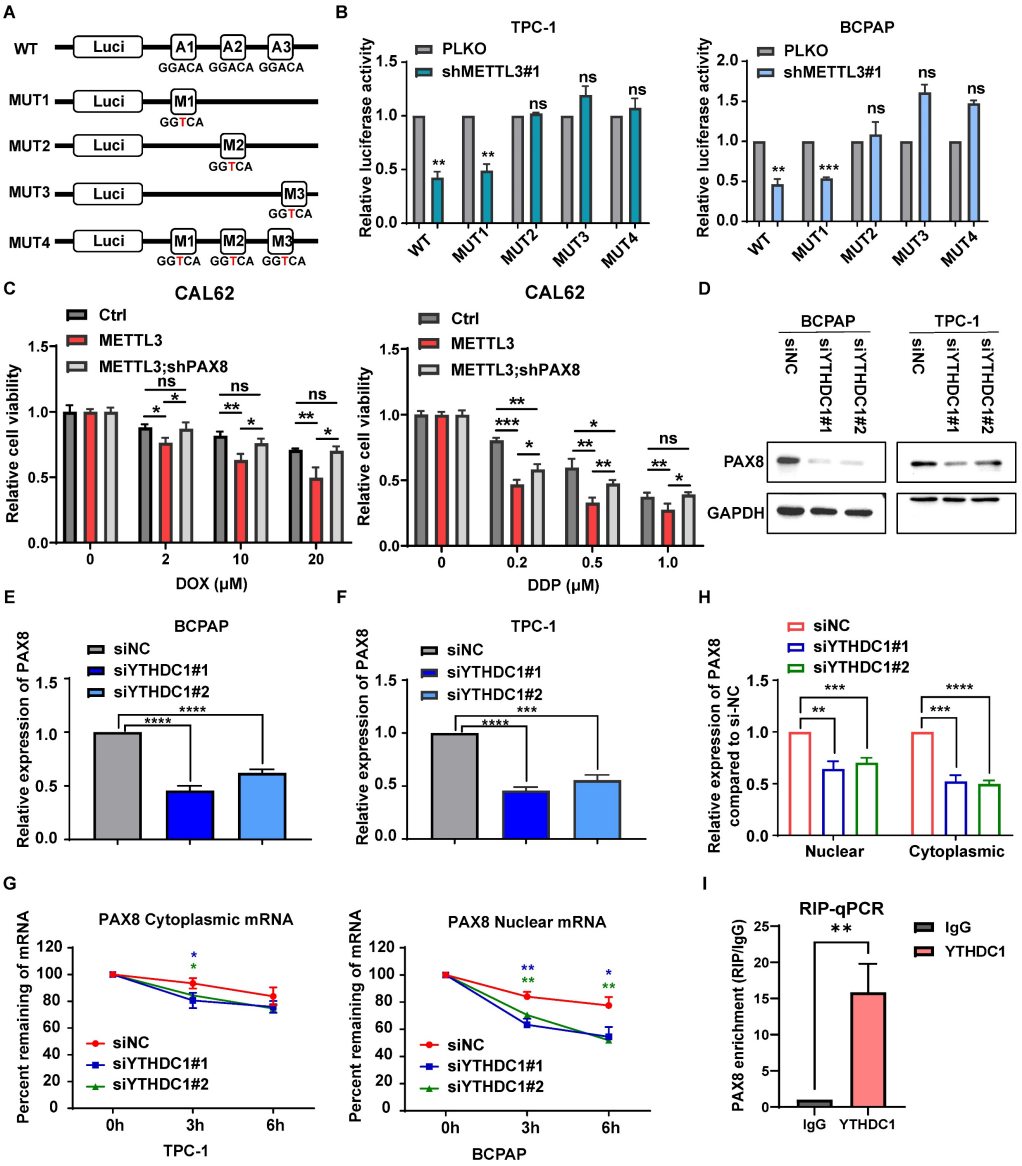
** METTL3 regulates the expression of PAX8 through m^6^A modification in thyroid carcinoma. (A)** The PAX8 3' UTR containing different point mutations was cloned into the pmirGlo dual-luciferase reporter. **(B)** The luciferase activity of different reporters that transfected into BCPAP and TPC-1 cell lines. **(C)** Relative cell viability of CAL-62 cells which transfected with PCDH METTL3 and shPAX8 treated with doxorubicin or cisplatin in different concentration gradients for 48 h. **(D)** The protein expression levels of PAX8 were examined by western blotting in siYTHDC1-transfected TPC-1 cells and BCPAP cells. **(E&F)** The RNA expression levels of PAX8 were examined by quantitative real-time PCR in siYTHDC1-transfected TPC-1 cells and BCPAP cells. **(G)** RT‒qPCR analysis of PAX8 mRNA expression levels in the cytoplasm and nucleus of control and YTHDC1 knockdown BCPAP cells. **(H)** Control and YTHDC1 knockdown BCPAP cells were treated with actinomycin D (5 μg/mL) for 0, 3, and 6 h. The expression levels of PAX8 mRNA in the cytoplasm and nucleus were analysed by RT‒qPCR. **(I)** The mRNA of PAX8 was co-incubate with YTHDC1 antibody and IgG, and the RNA obtained was subjected to RT-qPCR assay. Data represents mean ± SEM, *p< 0.05, **p < 0.01, ***p < 0.001, ****p < 0.0001.

To detect whether PAX8 is a direct downstream target gene of METTL3, we performed bioinformatic analysis using data from the TCGA database and observed that the expression of PAX8 was positively correlated with METTL3 (Fig. 4H). MeRIP experiments were further used to prove that the level of m^6^A modification on PAX8 mRNA increased upon METTL3 overexpression, which was further enhanced by coexpression of METTL3 and METTL14 (Fig. 4I). These findings demonstrated that METTL3 mediated m^6^A methylation modification of PAX8. To further clarify the role of METTL3 in regulating m^6^A modification of PAX8, we mutated three potential modification sites in PAX8, as predicted by SRAMP (http://www.cuilab.cn/sramp) (Supplementary Fig. 2F)^23^. The PAX8 3′ UTR containing different point mutations was cloned into the pmirGlo dual-luciferase reporter (Fig. 5A). The results showed that depletion of METTL3 significantly decreased the luciferase activity of PAX8 compared with that in the control group (Fig. 5B). Conversely, MUT2 and MUT3 increased PAX8 luciferase activity in TC cells with lower METTL3 expression, indicating that the GGTAC sequence in the 3′ UTR of PAX8 was essential for METTL3-mediated m^6^A regulation. These data together suggested that METTL3 directly regulates PAX8 by increasing its m^6^A methylation modification and transcription.

To validate PAX8 as the major downstream target of METTL3 in TC, we depleted PAX8 under conditions of METTL3 overexpression. The interference efficiency of siPAX8 was verified by qPCR and western blotting (Supplementary Fig. 3A-B). The results showed that depletion of PAX8 markedly attenuated the METLL3 overexpression-induced reduction in cell migration (Supplementary Fig. 3D), proliferation and viability (Supplementary Fig. 3E-F). We simultaneously overexpressed PAX8 (Supplementary Fig. 4A) in cell lines that knock down METTL3-expressing and performed cell cloning experiments, CCK8 assays and transwell experiments. The results showed that the overexpression of PAX8 could restore the effect of downregulation of METTL3 on cell proliferation, viability and migration. (Supplementary Fig. 4A-E). Similarly, knockdown of PAX8 can rescue the increased chemosensitivity caused by high expression of METTL3 (Supplementary Fig. 3C, Fig. 5C).

### The m^6^A reader protein YTHDC1 regulates PAX8 expression

To study the mechanism of PAX8 regulation by METTL3, we analysed the correlation between PAX8 and the m^6^A reader proteins that have been reported in the TCGA database (Supplementary Fig. 5A). YTHDC1 and YTHDC2 had the highest correlation with PAX8 and were selected as the research targets. Therefore, small interfering RNAs of YTHDC1 (siYTHDC1) and YTHDC2 (siYTHDC2) were transfected into the BCPAP and TPC-1 cells (Supplementary Fig. 5B). The western blotting and qPCR results showed that the interference of YTHDC1 could downregulate the expression of PAX8 (Fig. 5D-E), while YTHDC2 had no significant effect on the expression of PAX8 (Supplementary Fig. 5C-D). We speculated that YTHDC1, a m^6^A reader protein that promotes the export of m^6^A-methylated mRNAs from the nucleus^24^, ^25^ and regulates the stability of nuclear mRNA^26^, is the reader of METTL3-mediated PAX8 m^6^A. In addition, both nuclear and cytoplasmic PAX8 mRNA levels were reduced upon YTHDC1 depletion (Fig. 5G). Intriguingly, knockdown of YTHDC1 decayed PAX8 mRNA in the nucleus but not in the cytoplasm (Fig. 5H). These data reveal that YTHDC1 plays an important role in maintaining the stability of PAX8 mRNA modified by m^6^A in the nucleus. In order to investigate whether YTHDC1 indirectly affects or directly binds to PAX8, we constructed a stable cell line overexpressing YTHDC1 and proved that YTHDC1 binds directly to PAX8 mRNA by RIP-qPCR (Supplementary Fig. 5E; Fig. 5I). Morever, we found that YTHDC1 can promote the malignant behavior of thyroid cancer, such as proliferation and migration ability, through colony formation assays, CCK8 assays, and transwell assays (Supplementary Fig. 6A-E).

### miR-493-5p targeted METTL3 and inhibited its expression in TC cells

**Figure 6:**
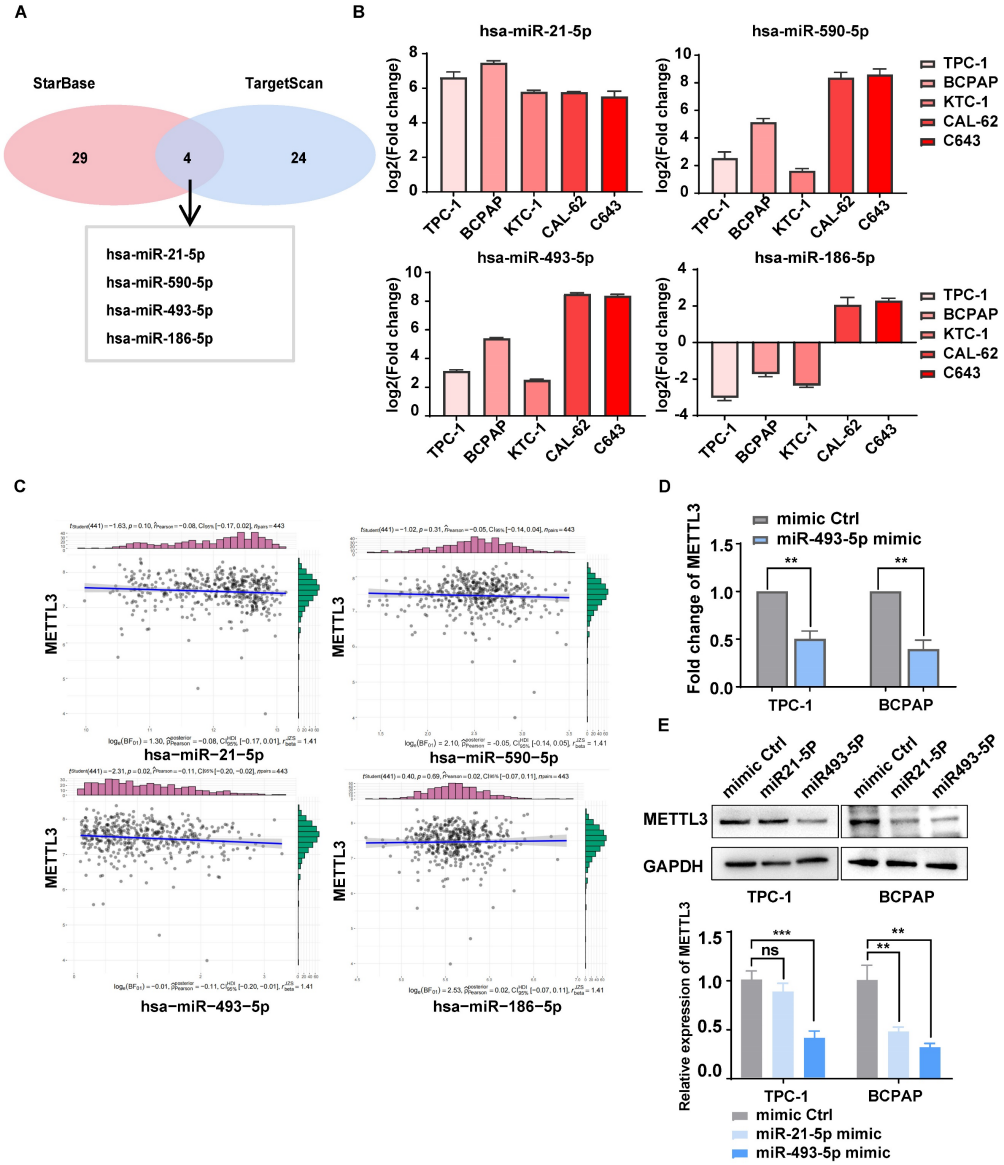
** miR-493-5p targeted METTL3 and inhibited its expression in TC cells. (A)** The microRNAs that can downregulate METTL3 were mined from TargetScan and starBase.** (B)** The RNA expression levels of microRNAs were examined by quantitative real-time PCR in normal thyroid cells and TC cells. **(C)** The correlation between METTL3 and these miR-RNAs was analysed by using the data of TC patients in the TCGA database. **(D)** The RNA expression levels of METTL3 were examined by quantitative real-time PCR in miR-493-5p mimic-transfected TPC-1 cells and BCPAP cells. **(E)** The protein expression levels of METTL3 were examined by western blotting in miR-21-5p mimic- and miR-493-5p-transfected TPC-1 cells and BCPAP cells. Data represents mean ± SEM, *p< 0.05, **p < 0.01, ***p < 0.001.

METTL3 is expressed at lower levels in TC tissue than in normal thyroid tissue^9^, ^27^, but the underlying mechanism is still unclear. Interestingly, four miRNAs that may degrade METTL3 were mined from databases and predicted by TargetScan and starBase (Fig. 6A). These miRNAs were detected in PTC, ATC and NTHY cell lines by RT‒qPCR (Fig. 6B). The correlation between METTL3 and these miR-RNAs was further analysed by using the data of TC patients from the TCGA database, and miR-493-5p showed a strong correlation with METTL3 (Fig. 6C). Notably, the addition of the miR-493-5p mimic significantly inhibited the mRNA and protein expression of METTL3 in TPC-1 and BCPAP cells (Fig. 6D-E). Through CCK8, colony formation assays, and transwell assays, we found that miR-493-5p mimic could rescue the inhibitory effect of METTL3 on the malignant behavior of thyroid cancer cells, including proliferative and migration ability (Supplementary Fig. 6F-I). Therefore, we concluded that miR-493-5p could reduce the expression of METTL3 in TC cells.

### METTL3 effects on the TC progression and sensitivity to chemotherapeutics dependent on PAX8 *in vivo*

**Figure 7:**
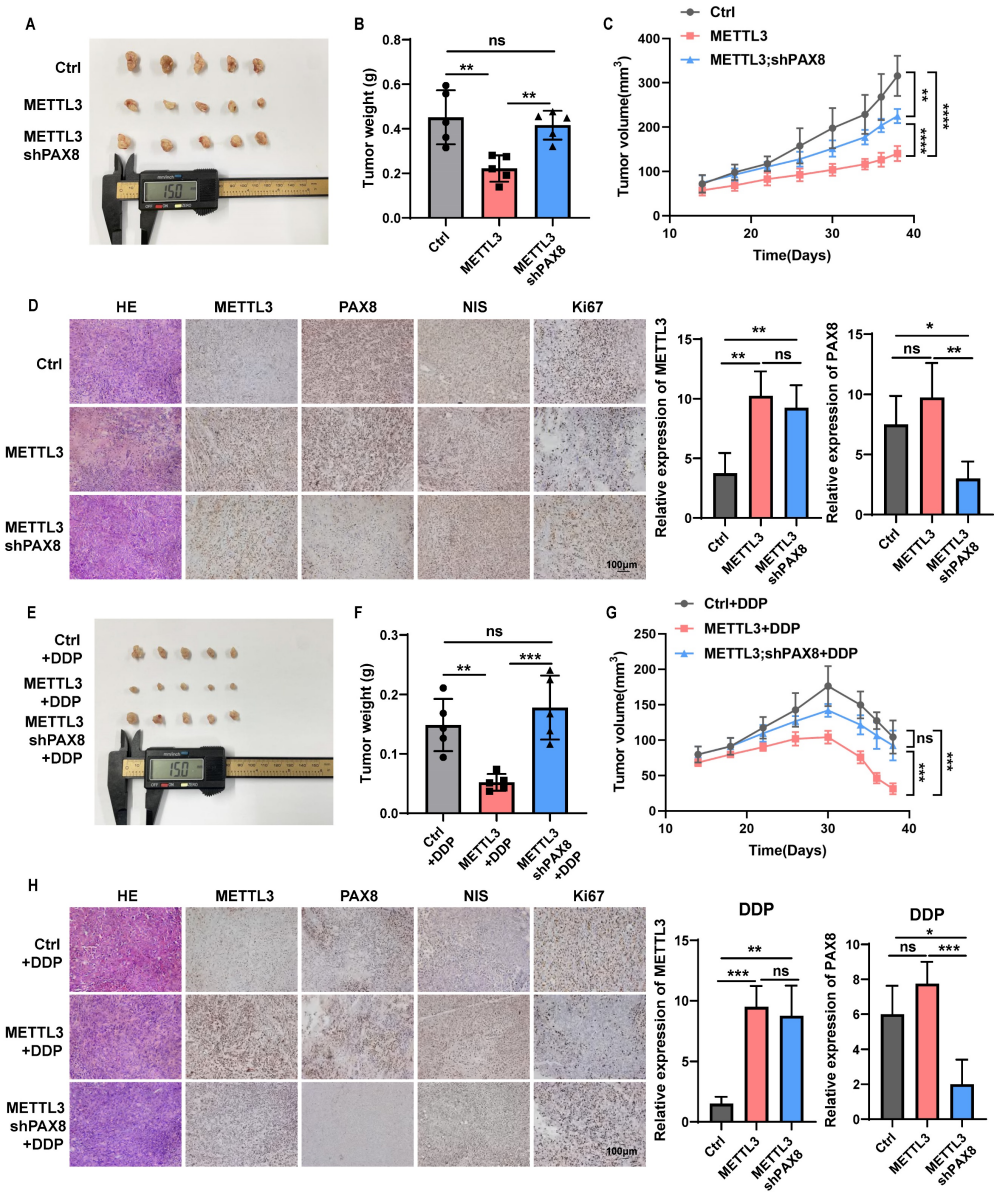
** METTL3 effects on the TC progression and sensitivity to chemotherapeutics dependent on PAX8 *in vivo*. (A)** Representative images of subcutaneous xenografts in nude mice derived from PCDH-transfected CAL-62 cells and OE METTL3-transfected CAL-62 cells or OE METTL3;shPAX8-transfected CAL-62 cells. **(B)** Analysis of the tumour weight of the xenografts in each group. **(C)** Growth curves of the subcutaneous xenografts in each group. **(D)** The expression levels of METTL3, PAX8, NIS and Ki67 in xenografts of each group were assessed by immunohistochemical staining. The scale bar is 100 μm. On the right side of the IHC photo are charts of the IHC score of METTL3 and PAX8. **(E)** Representative images of subcutaneous xenografts in nude mice derived from PCDH-transfected CAL-62 cells and OE METTL3-transfected CAL-62 cells or OE METTL3;shPAX8-transfected CAL-62 cells. These tumors were treated with DDP after implantation. **(F)** Analysis of the tumour weight of the xenografts in each group. **(G)** Growth curves of the subcutaneous xenografts in each group. **(H)** The expression levels of METTL3, PAX8, NIS and Ki67 in xenografts of each group were assessed by immunohistochemical staining. The scale bar is 100 μm. On the right side of the IHC photo are charts of the IHC score of METTL3 and PAX8. Data represents mean ± SEM, *p< 0.05, **p < 0.01, ***p < 0.001, ****p < 0.0001.

Finally, we further determined whether METTL3 mediates malignant behavior of thyroid cancer cells dependent on PAX8 *in vivo*. CAL-62 cells overexpressing METTL3 and cells that not only overexpress METTL3 but also knock down PAX8 were subcutaneously injected into mice to trigger TC tumorigenesis. Significantly, the down-regulate of PAX8 can reverse the inhibitory effect of METTL3 on the proliferative ability of thyroid cancer (Fig. 7A-D; Supplementary Fig. 7A). Furthermore, we found METTL3 could also significantly enhanced the sensitivity of mouse subcutaneous tumours to DDP or DOX treatment, resulting in tumour growth retardation (Fig. 7E-H; Supplementary Fig. 7B-G).

In addition, we found that METTL3 expression not only increased the levels of PAX8 but also inhibited the expression of NIS in tissue samples, which indicates that METTL3 relies on the expression of PAX8 to increase the sensitivity to chemotherapy drugs for thyroid cancer. It can also promote the expression of NIS, which may also be an important reason for improving the sensitivity of thyroid cancer radiotherapy and chemotherapy (Supplementary Fig. 7A-B, G).

## Discussion

m^6^A RNA methylation plays important roles in various physiological and pathological processes, including tumorigenesis^28^. Moreover, a previous study demonstrated that low expression of METTL3 was associated with a poor prognosis using IHC data from 58 PTC specimens^9^. These results were similar to the results we obtained in the GEO databases, which showed the expression levels of METTL3 in PTC tissue were lower than those in normal thyroid tissue. In addition, METTL3 expression is significantly different between TC and normal tissues. In addition, we performed IHC staining according to the pathological classification of TC, including normal thyroid (24), PTC (75), PDTC (26) and ATC stages (17). Surprisingly, the results showed that the expression level of METTL3 decreased significantly with decreasing TC differentiation. The expression difference in ATC is relatively large, and the number of cases we studied was limited, which may be the reason why there was no statistical significance when ATC was compared with PDTC. Our results suggest that METTL3 expression may be related to the dedifferentiation of TC.

To date, there has been no report on the relationship between METTL3 expression and prognosis of clinical TC patients. Here, we collected clinical information from approximately 142 patients. The analysis results suggested that low METTL3 expression was positively correlated with advanced T stage, lymph node metastasis and AJCC-defined stage of extrathyroidal extension. Moreover, genetically engineered* Braf^V600E^* mouse models were used in our study^11^, ^16^. It has been reported that *Braf^V600E^* leads to malignant transformation of thyroid epithelial cells into PTC cells, which rapidly progresses to DTC in mice^10^. In the present study, we investigated the cancer-promoting effect of METTL3 silencing in *Braf^V600E^* knock-in mice. We were surprised to find that *METTL3* knockout mice had lower differentiation of TC and a higher probability of lung metastasis. In addition, the loss of METTL3 expression also shortened their survival period.

This study on the biological function of METTL3 showed that METTL3 deficiency accelerates PTC cell proliferation and metastasis both *in vitro* and *in vivo*. However, the scope was limited to PTC^9^, ^29^. It has been proven that the degree of tumour differentiation affects chemosensitivity. In our study, we found that the overexpression of METTL3 enhanced the chemosensitivity of TC cells in both PTC and ATC cells, suggesting that METTL3 might be an important tumour suppressor gene and provide novel approaches for potential therapeutics.

Here, using high-throughput RNA-seq, we identified that the downregulation of METTL3 had a great impact on the formation and biological function of the cell membrane. In addition to the results we observed *in vivo*, we were very interested in genes related to TC differentiation. PAX8, TG, TPO, TSHR and NKX2.1 are key thyroid transcription factors that play important roles in the development of the thyroid^30^. The results of our research also showed that the low expression of METTL3 reduced the expression levels of PAX8, TG, TPO, TSHR and NKX2.1. In addition, NIS is an essential protein in thyroid tissue that can be regulated by PAX8^31^. We also evaluated its expression level using RT‒qPCR and western blotting. Notably, the downregulation of METTL3 decreased the expression level of NIS. This finding showed that the decreased expression of METTL3 leads to the downregulation of PAX8, which in turn affects the expression of NIS, which may be the underlying mechanism of TC progression and dedifferentiation. MeRIP experiments confirmed that the regulation of m^6^A modification on PAX8 is dependent on METTL3 expression.

At present, no m^6^A reader protein related to PAX8 has been reported. Here, we have identified that YTHDC1 is a reader protein of PAX8, which facilitates the transport of PAX8 from the nucleus to the cytoplasm. It was reported that several microRNAs, including miR-186, miR-4429, miR-600 and let-7 g were proposed to suppress METTL3 by targeting METTL3 mRNA^32^-^34^. We found high expression of miR-493-5P primarily downregulated METTL3 expression in TC cells.

In summary, our study reveals that the downregulated expression of METTL3 reduces the m^6^A modification level of PAX8. YTHDC1 recognize the m^6^A modification of PAX8 and promote the transport of PAX8 from the nucleus to the cytoplasm. The expression of PAX8 promote the differentiation of TC, and thus affects the expression of NIS. In addition, we also presented evidence that METTL3 is a direct target of miR-493-5P. Therefore, we introduce miR-493-5P and METTL3 as novel candidate markers for PTC diagnosis and therapy. These findings provide new treatment targets that have potential for application in the clinical treatment of RAIR-DTC.

## Materials and Methods

### Clinical samples

From January 2013 to March 2022, formalin-fixed paraffin-embedded (FFPE) samples of TC and normal tissue and frozen samples of TC tissue were obtained from the Cancer Hospital of Tianjin Medical University. All of these samples were examined by experienced pathologists who confirmed the diagnosis of the disease. Clinicopathological data, including sex, age, tumour size, lymph node metastasis, number of tumour lesions, haematological examination, glandular invasion and other clinicopathological characteristics, were collected. Tumour, node, metastasis (TNM) staging was based on the AJCC 8th Edition Differentiated Thyroid Cancer TNM staging criteria. The study was conducted under the approval of the Institutional Review Board of Tianjin Medical University Cancer Institute and Hospital.

### Cell lines and cell culture

Four PTC cell lines (BCPAP, KTC-1, TPC-1, K-1), two ATC cell lines (CAL-62 and C643) and a normal thyroid follicular epithelial cell line (Nthy-ori 3-1) were used in this study. HEK293T, TPC-1, K-1 and Nthy-ori 3-1 cells were purchased from the American Type Culture Collection (ATCC, USA), and the other cell lines were purchased from the Type Culture Collection of the Chinese Academy of Sciences (Shanghai, China). The cells in the experiment were all identified by short tandem repeat DNA profiling analysis, and they were all cultured within 20-30 generations, and there was no contamination of Mycoplasma. All the cells were maintained at 37°C in a 5% CO2 cell culture incubator.

### Gene expression in TC data sets

GEO datasets (GSE3467^12^, GSE33630^13^, ^14^, and GSE76039^15^) were used to validate METTL3 expression and its correlation with the pathological differentiation degree in TC patients.

### RNA extraction and RT-PCR

TRIzol Reagent (Invitrogen, USA) was used to extract total RNA from tissues or cell lines according to the manufacturer's instructions. The total RNA was reverse transcribed to cDNA using a Reverse Transcription Kit (Takara, Dalian, China). Specific primers and SYBR Premix Ex Taq II (Takara, Dalian, China) were used to detect the mRNA level of the target gene on the basis of the abovementioned cDNA as a template. All samples were normalized to β-actin or 18S rRNA levels. The primers used in RT‒qPCR are listed in Supplementary Table 2.

### Immunohistochemistry, scoring and HE staining

The protein expression level was evaluated by immunohistochemical staining of FFPE samples according to standard methods. The antibodies used were as follows: anti-METTL3 (Abcam, ab195352), anti-PAX8 (Abcam, ab239363), anti-NIS (Thermo Fisher Scientific, MA5-12308), and anti-Ki67 (CST, #9449). The immunoreactive score was produced by multiplying the percentage of positive cells and staining intensity. In detail, the percentage was scored as follows: nonpositive cells as 0 points, 1-30% as 1 point, 31-60% as 2 points, 61-80% and 81-100% as 3 and 4 points, respectively. The intensity of staining was defined as follows: no positive staining as 0 points, weak staining as 1 point, moderate staining as 2 points, and strong staining as 3 points. The final score was obtained as four grades: 0 points indicated no expression, 1-3 indicated low expression, 4-8 indicated moderate expression, and 9-12 indicated high expression. Sections were also stained with haematoxylin and eosin (HE).

### Mice

Animals used in this study were of specific-pathogen free (SPF) grade. The *TPO-Cre* and *LSL-Braf^V600E^CA* mice used in this study were from Professor Li Zhao's laboratory at Tianjin Medical University, and the *METTL3^flox/flox^* mice were purchased from Shanghai Model Organisms Center. All experimental mice were raised in a SPF environment in the experimental animal centre of Tianjin Medical University. All experimental operations were in accordance with the guidelines of the experimental animal welfare committee of Tianjin Medical University. Tumour tissues were obtained at 5 weeks to analyse tumour progression.

### Protein extraction and western blot analysis

Proteins from fresh tissues and cell lines were extracted with RIPA buffer containing protease inhibitors and phosphatase inhibitors. The protein concentration was determined by the BCA method, and the proteins were separated by 8%-12% SDS-PAGE, transferred to PVDF membranes, and blocked in 5% nonfat milk or BSA in TBS/Tween-20. The membr anes were incubated overnight at 4°C in the corresponding antibodies, including anti-METTL3 (Abcam, ab195352), anti-PAX8 (Abcam, ab239363), anti-NIS (Thermo Fisher Scientific, PA5-104351), anti-GAPDH (Proteintech, 60004-1-Ig), and anti-YTHDC1 (CST, 87459S). Subsequently, luminescence detection was performed using ECL reagents after incubation with the corresponding secondary antibodies.

### Plasmids and lentiviruses

For shRNA plasmids used in lentivirus-mediated interference, complementary sense and antisense oligonucleotides encoding shRNAs targeting METTL3 were synthesized, annealed and cloned into the pLKO.1 vector. The plasmids for expressing METTL3 were generated by inserting the cDNA into a pCMV vector. For lentivirus production, lentiviral vectors were cotransfected into HEK293T cells with the packaging vectors psPAX2 (#12260, Addgene) and pMD2. G (#12259, Addgene) using Lipofectamine 2000 (Invitrogen, Shanghai, China) according to the manufacturer's instructions. Infectious lentivirus particles were harvested at 48 hours after transfection, filtered through a 0.45 μM filter unit (Millipore) and transduced into TC cells. 1 μg/ml puromycin was added to the culture medium to screen stable cell lines. siRNAs were transfected into cells using Lipofectamine 2000. The cells were harvested for further experiments after 48 hours. The plasmids for expressing YTHDC1 and PAX8, shRNAs targeting PAX8 and all siRNAs were synthesized by Hanbio company. The related sequences of shRNA and siRNA are shown in Supplementary Tables 3 and 4.

### Cell viability and colony formation

Cells were plated in quadruplicate in 96-well plates at 1000 cells per well and cultivated with media supplemented with 10% FBS. Cell viability was assessed using the Cell Counting Kit-8 (Meilunbio, MA0218). The optical density (OD) was read on a Bio Tek Eon Multi-Mode Microplate Reader. Cell growth curves were constructed by GraphPad Prism 7.0. For the colony formation assay, cells were plated in 6-well plates at a density of 1000 cells per well and cultured for 10 days. Cells were fixed with 4% paraformaldehyde for 2 h. The cells were then stained with crystal violet for 30 minutes. The colonies were manually counted.

### RNA dot blot

Total RNA was extracted by TRIzol reagent (Invitrogen, USA) following the manufacturer's instructions, followed by the purification of polyadenylated mRNA employing Dynabeads mRNA purification kits (Invitrogen) according to the manufacturer's instructions. Poly(A) RNA was denatured at 65°C for 5 minutes and spotted onto a nylon membrane (GE Healthcare, USA), followed by UV crosslinking at UV 254 nm, 0.12 J/cm2. Then, the sections were stained with 0.02% methylene blue (Sangon Biotech, China), followed by scanning to indicate the total content of input RNA. After being blocked with 5% nonfat milk, the membrane was incubated with a specific m^6^A antibody (1:1000, Millipore) overnight at 4 °C. Dot blots were reacted with HRP-conjugated anti-mouse immunoglobulin G (IgG) for 1 h before visualization by an imaging system (Bio-Rad, USA).

### RNA-seq analysis

BCPAP cells were lysed using TRIzol reagent (Invitrogen, USA) after transfection with shMETTL3, and stable expression was verified. The global gene expression profiles were detected by mRNA sequencing from LC-BIO Biotech (Hangzhou, China). Bioinformatics analyses were implemented as previously described.

### MeRIP-qPCR

A portion (10%) of RNA was obtained as an input sample. RNA was immunoprecipitated with anti-m^6^A antibody (Abcam) in immunoprecipitation buffer (150 mM NaCl, 10 mM Tris-HCl, pH 7.5, 0.1% IGEPAL CA-630, 1 U/l RNase inhibitor) at 4°C for 6 h and then incubated with Dynabeads protein A (Invitrogen, USA) at 4°C for 2 h. The bound RNA was eluted twice by competition with m^6^A 5′-monophosphate sodium salt at 4°C for 1 h. Following ethanol precipitation, the input RNA and immunoprecipitated m^6^A RNAs were determined by RT‒qPCR analysis.

### ^131^I uptake of thyroid carcinoma cells assay

Thyroid cancer cells in logarithmic growth phase were placed in 24-well plates at 1×10^5^ cells per well and cultured in a 37°C, 5% CO_2_ incubator for 24 hours. After washing with PBS, 185 kBq Na131I solution and 10 µM NaI were added, and the cells were cultured at 37°C for 30 minutes. The cells were washed with PBS, and anhydrous ethanol solution was added for 5 minutes for the reaction before using a γ-ray counting system to measure the radioactivity level for 60 seconds.

### Luciferase reporter assay

cDNAs containing partial CDS sequence near stop codon and full-length 3'UTR of PAX8 were cloned into pmirGLO-control vectors (Promega) which was comprised of firefly luciferase. For mutant 1, 2, 3 and 4 reporter plasmids, predicted adenosine (A) were replaced by cytosine (C), respectively. The inserted sequences were listed in Supplementary Table 5. Pre-treated TC cells were seeded into 24-well plate followed by co-transfection of 0.5µg of wild-type or mutated PAX8 reporter plasmids and 25 ng renilla luciferase reporter vector. After 24-36 h, cells were harvested to access the luciferase activity using Dual-Glo Luciferase system (Promega) with the normalization to pRL-TK. Each group was conducted in triplicate.

### Tumour xenografts

Five-week-old female BALB/c nude mice were purchased from Beijing Vital River Laboratory Animal Technology Company. Mice were randomly divided into control groups and different experimental groups by the random number method. A concentration of 1 × 10^7^ CAL62 cells stably transfected with OE METTL3 or OE METTL3/shPAX8-transfected CAL-62 cells were injected subcutaneously into the dorsal flanks of the mice (five mice per group) to establish a xenograft model. The nude mice were given the drug after one month. Xenografted mice were then administered DMSO, DDP (2 mg/kg per three days) or DOX (4 mg/kg per three days). Subcutaneous tumour sizes were measured every 3 days using Vernier callipers and calculated according to the following formula: Volume (mm^3^) = 1/2 × length × width^2^. The mice were sacrificed, and tumours were harvested and fixed with 4% paraformaldehyde after 10 days of treatment. The experimental protocol on nude mice was approved by the Ethics Committee of Tianjin Medical University Cancer Institute and Hospital. All procedures involving animals and their care were conducted in accordance with institutional guidelines from national and international institutions.

### Statistical analysis

All data are expressed as the mean ± SD of at least three separate experiments. Statistical significance was evaluated with a two-tailed paired t test by using SPSS 17.0 software and GraphPad Prism 7.0 software. A p value of <0.05 was considered to indicate significance.

## Supplementary Material

Supplementary figures and tables.

## Figures and Tables

**Figure 8:**
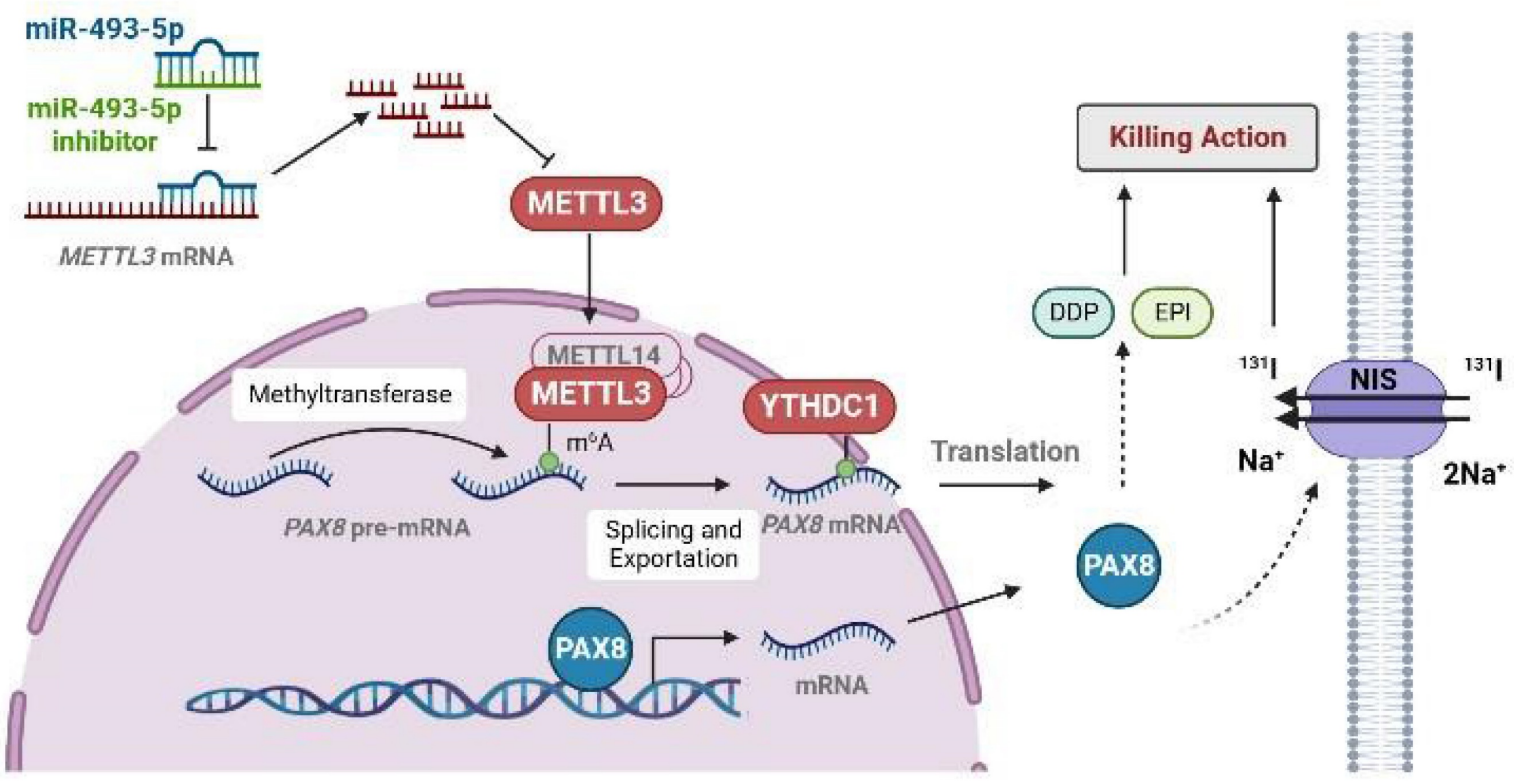
** The pattern diagram of METTL3 regulates the expression of PAX8 through m^6^A modification.** METTL3 added m^6^A on PAX8 pre-mRNA so that PAX8 can be recognized by YTHDC1 and promote PAX8 mRNA out of the nucleus. The expression of PAX8 could promote the expression of NIS on the cell membrane. In thyroid cancer cells, miR493-5P can recognize and promote METTL3 degradation to regulate the expression of PAX8.

## Abbreviations

TC: Thyroid carcinoma
METTL3: Methyltransferase-like 3
PTC: Papillary thyroid carcinoma
m^6^A: *N*6- methyladenosine
ATC: Anaplastic thyroid cancer
NIS: Na^+^/I^-^ symporter
^131^I: Iodine-131
DTC: Differentiated thyroid cancer
RAI: Radioactive iodine
TSH: Thyroid-stimulating hormone
PI3K: Phosphoinositide 3-kinase
MAPK: MAP kinase kinase kinase
PAX8: Paired box 8

## References

[1] Li M, Pei J, Xu M, Shu T, Qin C, Hu M (2021). Changing incidence and projections of thyroid cancer in mainland China, 1983-2032: evidence from Cancer Incidence in Five Continents. Cancer Causes Control.

[2] Durante C, Haddy N, Baudin E, Leboulleux S, Hartl D, Travagli JP (2006). Long-term outcome of 444 patients with distant metastases from papillary and follicular thyroid carcinoma: benefits and limits of radioiodine therapy. J Clin Endocrinol Metab.

[3] Liao Y, Hua Y, Li Y, Zhang C, Yu W, Guo P (2021). CRSP8 promotes thyroid cancer progression by antagonizing IKKalpha-induced cell differentiation. Cell Death Differ.

[4] Tang J, Tian Z, Liao X, Wu G (2021). SOX13/TRIM11/YAP axis promotes the proliferation, migration and chemoresistance of anaplastic thyroid cancer. Int J Biol Sci.

[5] Xing M (2013). Molecular pathogenesis and mechanisms of thyroid cancer. Nat Rev Cancer.

[6] Zaballos MA, Santisteban P (2017). Key signaling pathways in thyroid cancer. J Endocrinol.

[7] Kakun RR, Melamed Z, Perets R (2022). PAX8 in the Junction between Development and Tumorigenesis. Int J Mol Sci.

[8] Ruan X, Tian M, Kang N, Ma W, Zeng Y, Zhuang G (2021). Genome-wide identification of m6A-associated functional SNPs as potential functional variants for thyroid cancer. Am J Cancer Res.

[9] He J, Zhou M, Yin J, Wan J, Chu J, Jia J (2021). METTL3 restrains papillary thyroid cancer progression via m(6)A/c-Rel/IL-8-mediated neutrophil infiltration. Mol Ther.

[10] Knauf JA, Ma X, Smith EP, Zhang L, Mitsutake N, Liao XH (2005). Targeted expression of BRAFV600E in thyroid cells of transgenic mice results in papillary thyroid cancers that undergo dedifferentiation. Cancer Res.

[11] Charles RP, Iezza G, Amendola E, Dankort D, McMahon M (2011). Mutationally activated BRAF(V600E) elicits papillary thyroid cancer in the adult mouse. Cancer Res.

[12] He H, Jazdzewski K, Li W, Liyanarachchi S, Nagy R, Volinia S (2005). The role of microRNA genes in papillary thyroid carcinoma. Proc Natl Acad Sci U S A.

[13] Dom G, Tarabichi M, Unger K, Thomas G, Oczko-Wojciechowska M, Bogdanova T (2012). A gene expression signature distinguishes normal tissues of sporadic and radiation-induced papillary thyroid carcinomas. Br J Cancer.

[14] Tomas G, Tarabichi M, Gacquer D, Hebrant A, Dom G, Dumont JE (2012). A general method to derive robust organ-specific gene expression-based differentiation indices: application to thyroid cancer diagnostic. Oncogene.

[15] Landa I, Ibrahimpasic T, Boucai L, Sinha R, Knauf JA, Shah RH (2016). Genomic and transcriptomic hallmarks of poorly differentiated and anaplastic thyroid cancers. J Clin Invest.

[16] Franco AT, Malaguarnera R, Refetoff S, Liao XH, Lundsmith E, Kimura S (2011). Thyrotrophin receptor signaling dependence of Braf-induced thyroid tumor initiation in mice. Proc Natl Acad Sci U S A.

[17] Jing X, Xie M, Ding K, Xu T, Fang Y, Ma P (2022). Exosome-transmitted miR-769-5p confers cisplatin resistance and progression in gastric cancer by targeting CASP9 and promoting the ubiquitination degradation of p53. Clin Transl Med.

[18] Prawira A, Le TBU, Vu TC, Huynh H (2021). Ribociclib enhances infigratinib-induced cancer cell differentiation and delays resistance in FGFR-driven hepatocellular carcinoma. Liver Int.

[19] Hu W, Feng P, Zhang M, Tian T, Wang S, Zhao B (2022). Endotoxins Induced ECM-Receptor Interaction Pathway Signal Effect on the Function of MUC2 in Caco2/HT29 Co-Culture Cells. Front Immunol.

[20] Jia B, Yu S, Yu D, Liu N, Zhang S, Wu A (2021). Mycotoxin deoxynivalenol affects myoblast differentiation via downregulating cytoskeleton and ECM-integrin-FAK-RAC-PAK signaling pathway. Ecotoxicol Environ Saf.

[21] Stenzel D, Wilsch-Brauninger M, Wong FK, Heuer H, Huttner WB (2014). Integrin alphavbeta3 and thyroid hormones promote expansion of progenitors in embryonic neocortex. Development.

[22] Pasca di Magliano M, Di Lauro R, Zannini M (2000). Pax8 has a key role in thyroid cell differentiation. Proc Natl Acad Sci U S A.

[23] Zhou Y, Zeng P, Li YH, Zhang Z, Cui Q (2016). SRAMP: prediction of mammalian N6-methyladenosine (m6A) sites based on sequence-derived features. Nucleic Acids Res.

[24] Roundtree IA, Luo GZ, Zhang Z, Wang X, Zhou T, Cui Y (2017). YTHDC1 mediates nuclear export of N(6)-methyladenosine methylated mRNAs. Elife.

[25] Lesbirel S, Viphakone N, Parker M, Parker J, Heath C, Sudbery I (2018). The m(6)A-methylase complex recruits TREX and regulates mRNA export. Sci Rep.

[26] Liang D, Lin WJ, Ren M, Qiu J, Yang C, Wang X (2022). m(6)A reader YTHDC1 modulates autophagy by targeting SQSTM1 in diabetic skin. Autophagy.

[27] Lin S, Zhu Y, Ji C, Yu W, Zhang C, Tan L (2022). METTL3-Induced miR-222-3p Upregulation Inhibits STK4 and Promotes the Malignant Behaviors of Thyroid Carcinoma Cells. J Clin Endocrinol Metab.

[28] Jiang X, Liu B, Nie Z, Duan L, Xiong Q, Jin Z (2021). The role of m6A modification in the biological functions and diseases. Signal Transduct Target Ther.

[29] Zhu Y, Peng X, Zhou Q, Tan L, Zhang C, Lin S (2022). METTL3-mediated m6A modification of STEAP2 mRNA inhibits papillary thyroid cancer progress by blocking the Hedgehog signaling pathway and epithelial-to-mesenchymal transition. Cell Death Dis.

[30] Nilsson M, Fagman H (2017). Development of the thyroid gland. Development.

[31] Riesco-Eizaguirre G, Wert-Lamas L, Perales-Paton J, Sastre-Perona A, Fernandez LP, Santisteban P (2015). The miR-146b-3p/PAX8/NIS Regulatory Circuit Modulates the Differentiation Phenotype and Function of Thyroid Cells during Carcinogenesis. Cancer Res.

[32] Cui X, Wang Z, Li J, Zhu J, Ren Z, Zhang D (2020). Cross talk between RNA N6-methyladenosine methyltransferase-like 3 and miR-186 regulates hepatoblastoma progression through Wnt/beta-catenin signalling pathway. Cell Prolif.

[33] Wei W, Huo B, Shi X (2019). miR-600 inhibits lung cancer via downregulating the expression of METTL3. Cancer Manag Res.

[34] He H, Wu W, Sun Z, Chai L (2019). MiR-4429 prevented gastric cancer progression through targeting METTL3 to inhibit m(6)A-caused stabilization of SEC62. Biochem Biophys Res Commun.

