# Supplementary material for: METTL3 regulates thyroid cancer differentiation and chemosensitivity by modulating PAX8
Source: Int J Biol Sci. 2024 Jun 17;20(9):3426–41. doi: 10.7150/ijbs.84797 (PMC11234206; doi:10.7150/ijbs.84797)
Supplement: Supplementary file 1 — Supplementary figures and tables. [file ijbsv20p3426s1.pdf]

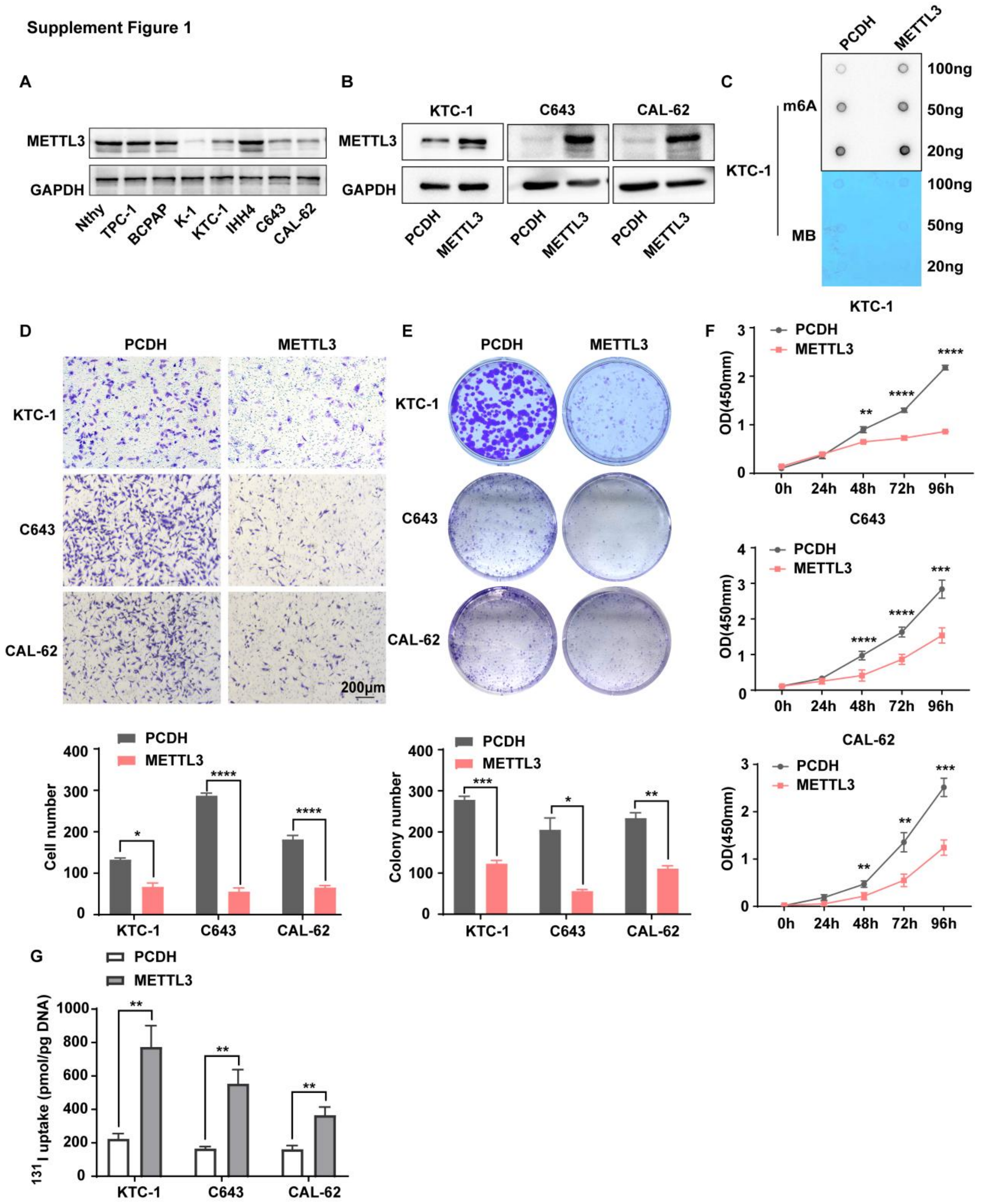

Supplemental Figure 1. (A) The protein expression levels of METTL3 were examined by western blotting in normal thyroid cells and TC cells. **(B)** The protein level of METTL3 was measured in METTL3-overexpressing KTC-1, C643, and CAL-62 cells by western blotting. **(C)** The level of m^{6}A modification in OE METTL3-transfected KTC-1 cells was measured by dot blot assay. **(D)** Transwell migration assays were used to detect the migration ability of

METTL3-overexpressing KTC-1, C643, and CAL-62 cells compared with control group. The statistical chart is below. **(E)** METTL3-overexpression plasmid-transfected KTC-1, C643, and CAL-62 cells were cultured for 14 days prior to crystal violet staining. The statistical chart is below. **(F)** The viability of METTL3-overexpressing plasmid-transfected KTC-1, C643, and CAL-62 cells compared with control cells was measured using the CCK-8 assay. **(G)** An iodine-131 cell uptake assay was used to measure iodine uptake in METTL3-overexpressing KTC-1, C643, and CAL-62 cells. Data are shown as the mean ± SD of three replicates. Data represents mean ± SEM, *$p < 0.05$, **$p < 0.01$, ***$p < 0.001$, ****$p < 0.0001$.

Supplement Figure 2

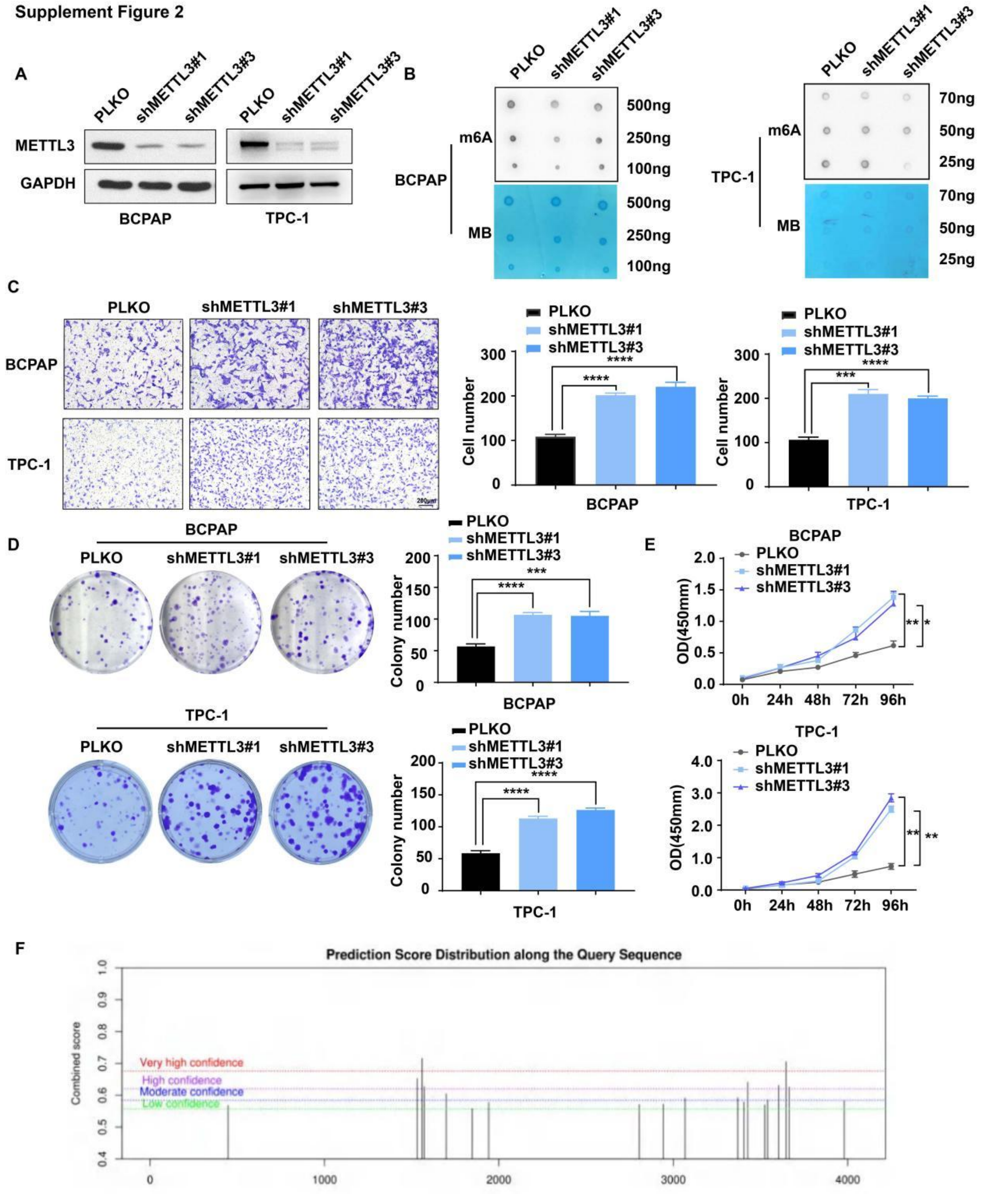

Supplemental Figure 2. (A) The protein level of METTL3 was measured in METTL3 knockdown BCPAP cells and TPC-1 cells by western blotting. **(B)** The level of m^{6}A modification in shMETTL3-transfected BCPAP cells and TPC-1 cells was measured by dot blot assay. **(C)** Transwell assays were used to measure the migration ability of METTL3 knockdown BCPAP cells and TPC-1 cells compared with control group. The statistical chart is on its right. **(D)**

BCPAP cells and TPC-1 cells which transfected with shMETTL3 vector were cultured for 14 days prior to crystal violet staining. **(E)** The viability of shMETTL3-transfected TPC-1 cells and BCPAP cells compared with control cells was measured using the CCK-8 assay. Data are shown as the mean ± SD of three replicates. **(F)** The potential modification sites of PAX8 induced by METTL3 were predicted by the SPRAMP website. Data represents mean ± SEM, $^{*}p < 0.05$, $^{**}p < 0.01$, $^{***}p < 0.001$, $^{****}p < 0.0001$.

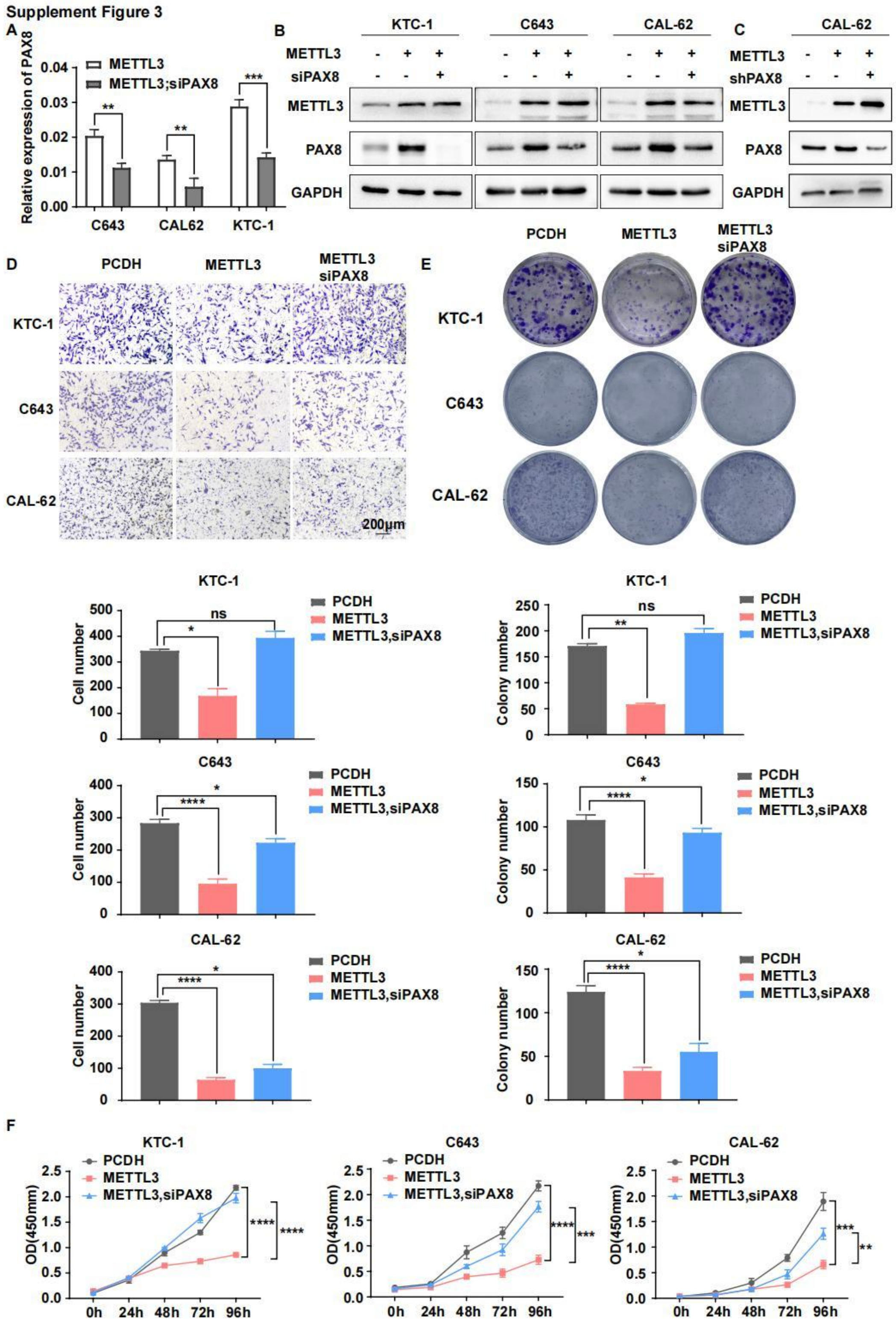

Supplement Figure 3. (A) The RNA expression levels of PAX8 were examined by quantitative real-time PCR in OE METTL3-transfected KTC-1, C643, and CAL-62 cells and siPAX8/OE METTL3-transfected KTC-1, C643,

and CAL-62 cells. **(B)** The protein expression levels of PAX8 were examined by western blotting in OE METTL3-transfected KTC-1, C643, and CAL-62 cells and siPAX8/OE METTL3-transfected KTC-1, C643, and CAL-62 cells. **(C)** A stable cell line which overexpressing METTL3 and knocking down PAX8 was constructed in CAL-62. **(D)** Transwell assays were carried out using KTC-1, C643, and CAL-62 cells stably overexpressing METTL3 and siPAX8-transfected KTC-1 cells or the corresponding negative control (NC) cells. **(E)** KTC-1, C643, and CAL-62 cells were stably transduced with OE METTL3 or siPAX8/OE METTL3 and cultured for 14 days prior to crystal violet staining. **(F)** The viability of OE METTL3-transfected KTC-1, C643, and CAL-62 cells and siPAX8-transfected KTC-1, C643, and CAL-62 cells compared with control group was measured using the CCK-8 assay. Data are shown as the mean ± SD of three replicates. Data represents mean ± SEM, *p< 0.05, **p < 0.01, ***p < 0.001, ****p < 0.0001.

Supplement Figure 4

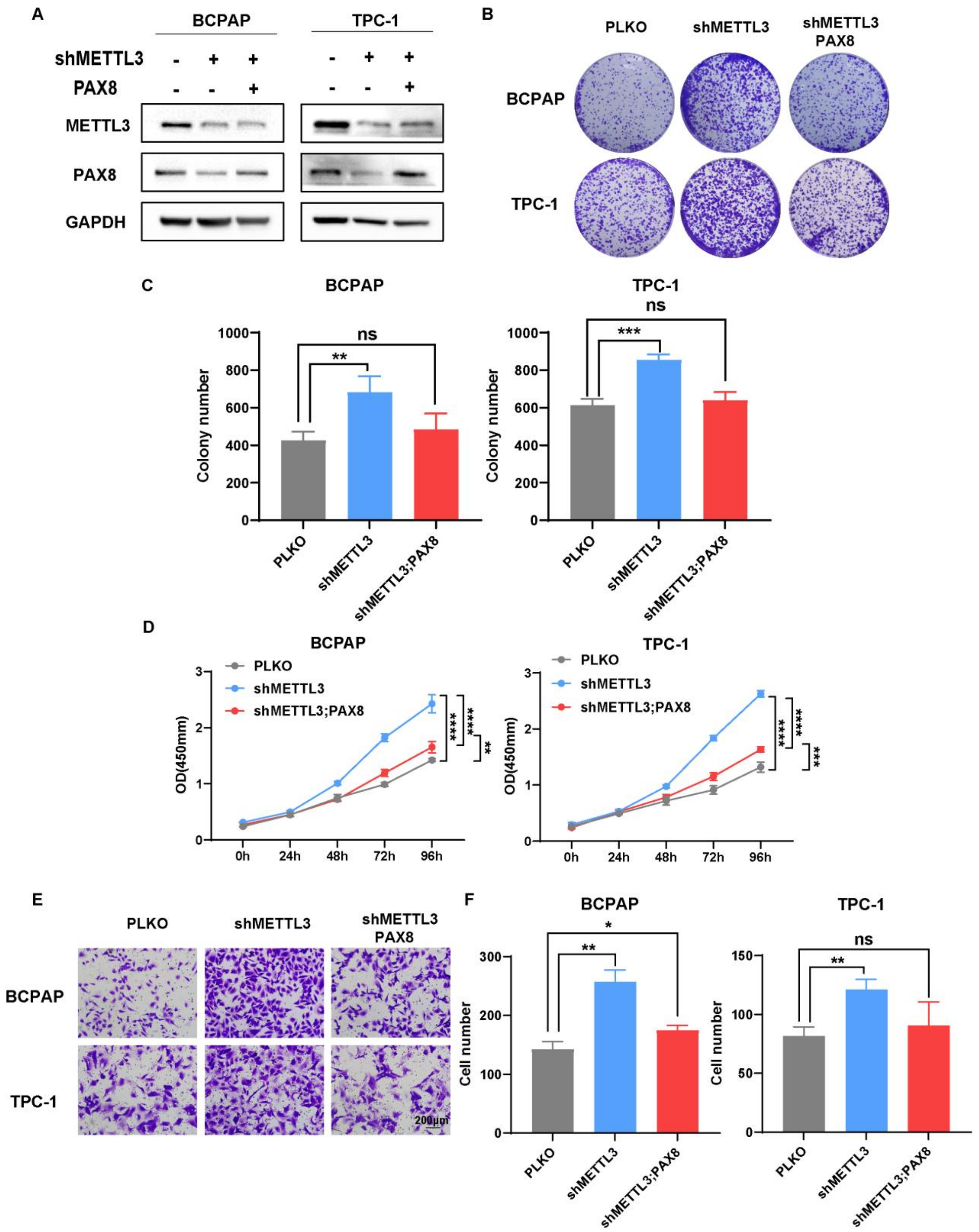

Supplement Figure 4. (A) The protein expression levels of METTL3 and PAX8 were examined by western blotting in shMETTL3-transfected BCPAP, TPC-1 cells and shMETTL3/OEPAX8-transfected BCPAP, TPC-1 cells. **(B)** BCPAP, TPC-1 cells were stably transduced with shMETTL3 or

shMETTL3/OEPAX8 and cultured for 14 days prior to crystal violet staining. **(C)** The statistical chart of colony formation assay. **(D)** The viability of shMETTL3-transfected BCPAP, TPC-1 cells and shMETTL3/OEPAX8-transfected BCPAP, TPC-1 cells compared with control group was measured using the CCK-8 assay. Data are shown as the mean ± SD of three replicates. **(E)** Transwell assays were carried out using BCPAP, TPC-1 cells that stably transduced with shMETTL3 or shMETTL3/OEPAX8. **(F)** The statistical chart of colony formation assay. Data represents mean ± SEM, *p< 0.05, **p < 0.01, ***p < 0.001, ****p < 0.0001.

.

Supplement Figure 5

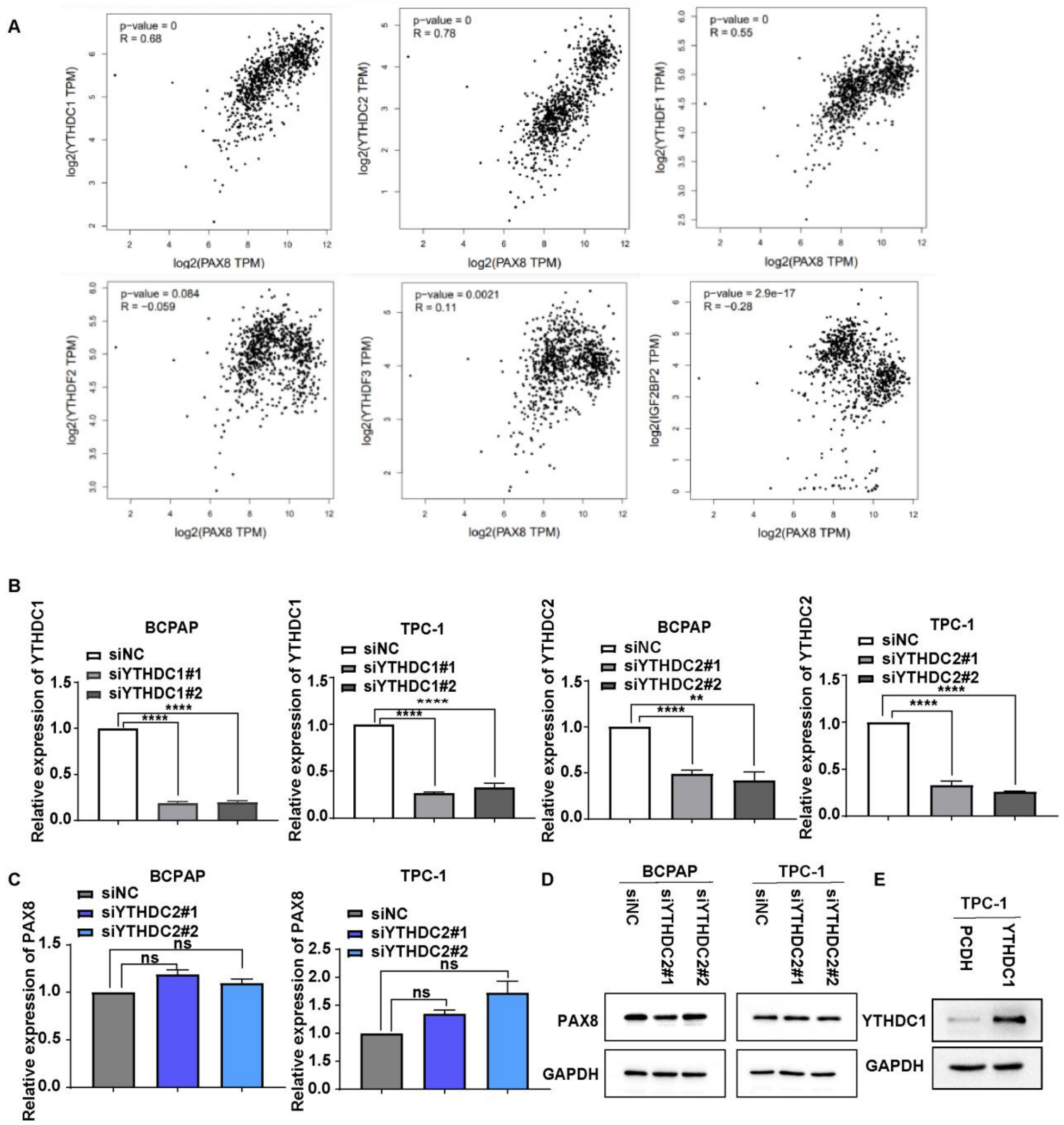

Supplemental Figure 5. (A) Bioinformatics analysis using data from the TCGA database showed that PAX8 expression was positively correlated with a number of m^{6}A reader proteins. **(B)** The RNA expression levels of YTHDC1 and YTHDC2 were examined by quantitative real-time PCR in siYTHDC1- and siYTHDC2-transfected BCPAP cells and TPC-1 cells. **(C)** The RNA expression levels of PAX8 were examined by quantitative real-time PCR in siYTHDC2-transfected BCPAP cells and TPC-1 cells. **(D)** The protein

expression levels of PAX8 were examined by western blotting in siYTHDC1- and siYTHDC2-transfected BCPAP cells and TPC-1 cells. **(E)** The cell line which stably overexpressing YTHDC1 were validated by western blotting. Data represents mean ± SEM, *$p < 0.05$, **$p < 0.01$, ***$p < 0.001$, ****$p < 0.0001$.

Supplement Figure 6

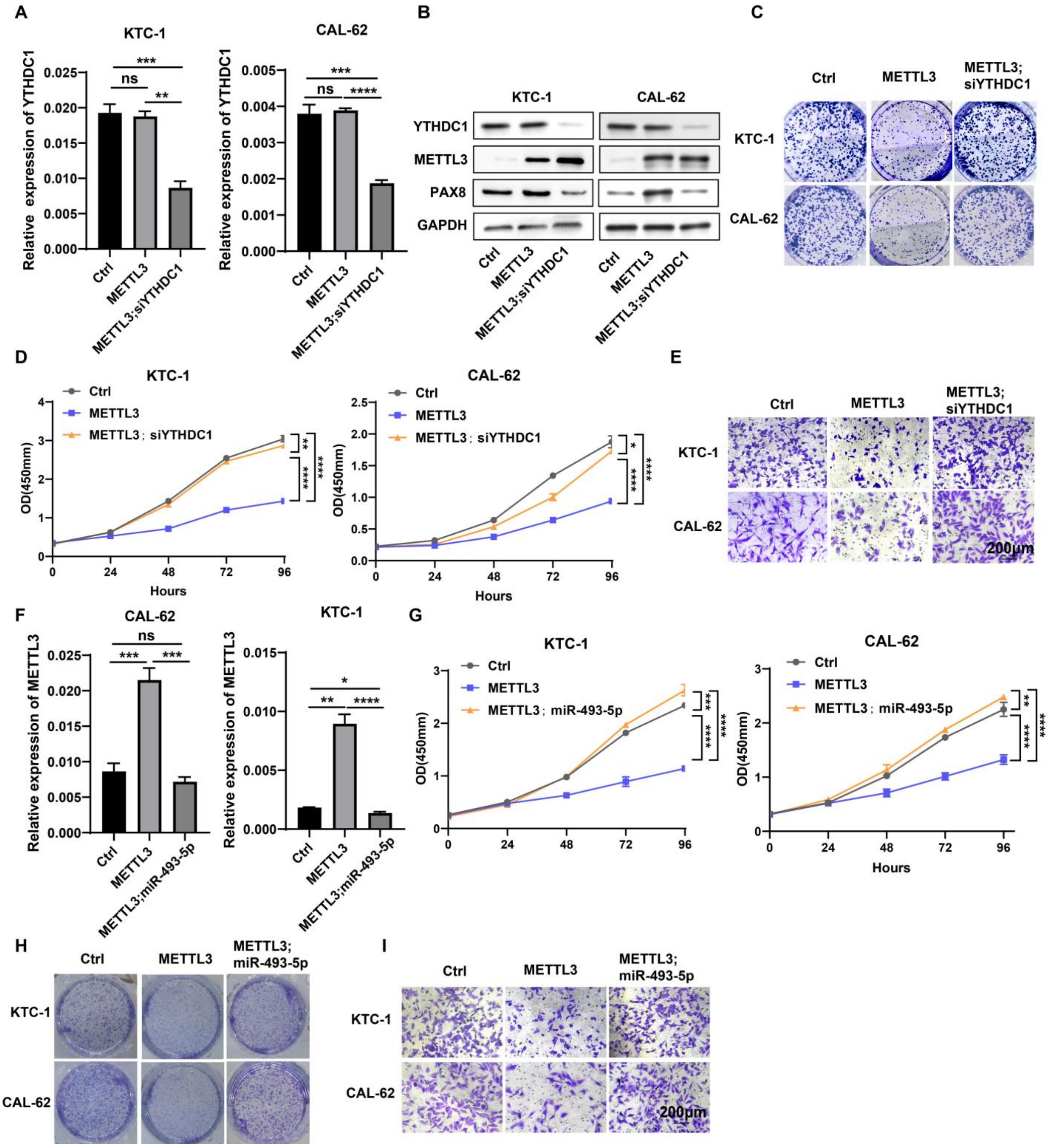

Supplement Figure 6. (A) The RNA expression levels of YTHDC1 were examined by quantitative real-time PCR in OE METTL3-transfected KTC-1, CAL-62 cells and siYTHDC1/OE METTL3-transfected KTC-1, CAL-62 cells. **(B)** The protein expression levels of YTHDC1 were examined by western blotting in OE METTL3-transfected KTC-1, CAL-62 cells and siYTHDC1/OE

METTL3-transfected KTC-1, CAL-62 cells. **(C-E)** Colony formation assays, CCK-8 assays and transwell assays performed by OE METTL3 or siYTHDC1/OE METTL3-transfected into KTC-1 and CAL-62 cells. **(F)** The RNA expression levels of METTL3 were examined by quantitative real-time PCR in OE METTL3-transfected KTC-1, CAL-62 cells and miR-493-5p mimic/OE METTL3-transfected KTC-1, CAL-62 cells. **(G-I)** CCK-8 assays， Colony formation assays and transwell assays performed by OE METTL3 or miR-493-5p mimic/OE METTL3-transfected into KTC-1 and CAL-62 cells. Data are shown as the mean ± SD of three replicates. Data represents mean ± SEM, *p< 0.05, **p < 0.01, ***p < 0.001, ****p < 0.0001.

Supplement Figure 7

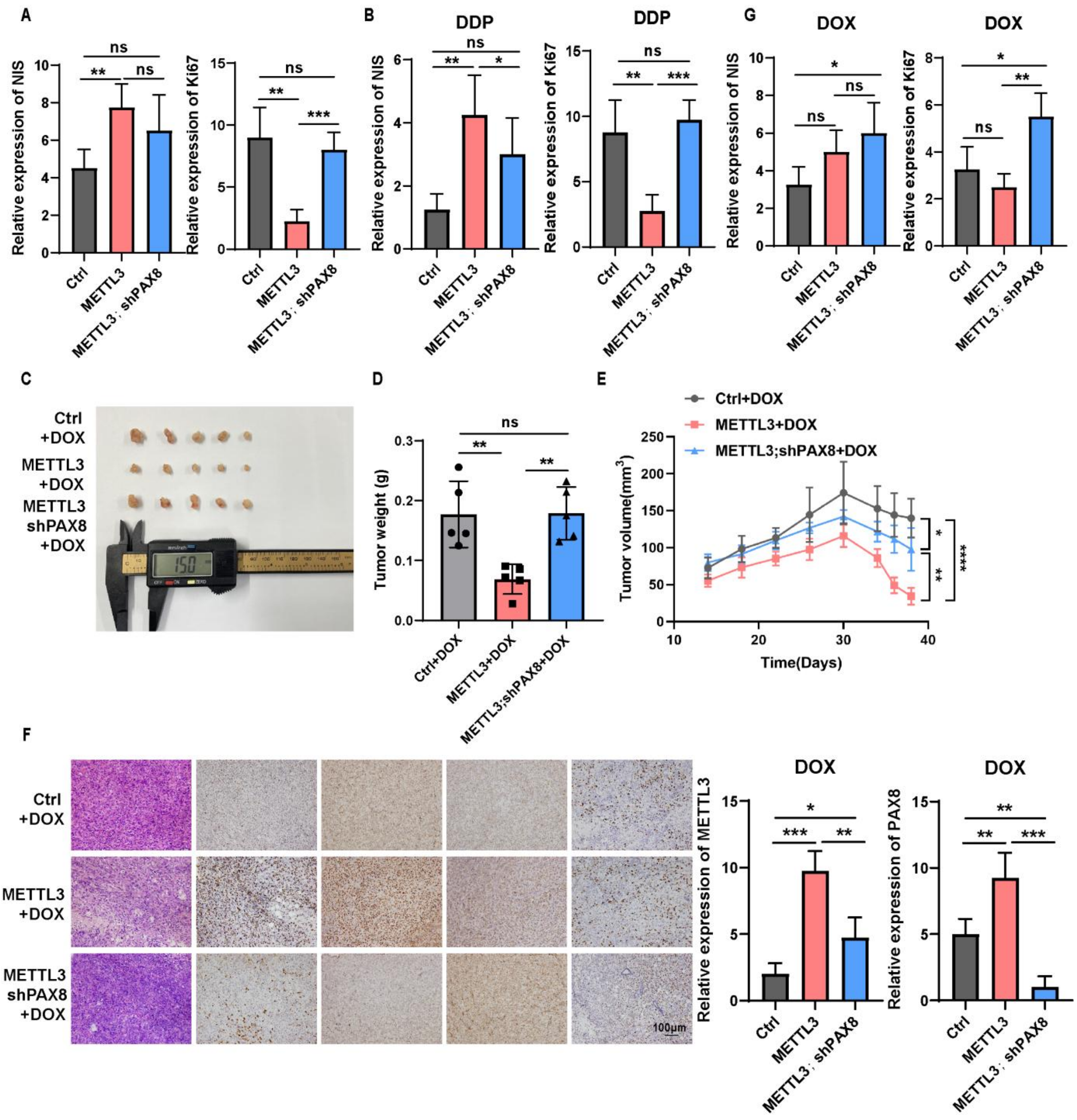

Supplement Figure 7. (A-B,G) Charts of the IHC score of NIS and Ki67 from subcutaneous xenografts in nude mice. Derived from PCDH-transfected CAL-62 cells and OE METTL3-transfected CAL-62 cells or OE METTL3/shPAX8-transfected CAL-62 cells, respectively. **(C)** Representative images of subcutaneous xenografts in nude mice derived from PCDH-transfected CAL-62 cells and OE METTL3-transfected CAL-62 cells or

OE METTL3/shPAX8-transfected CAL-62 cells. These tumors were treated with DOX after implantation. **(D)** Analysis of the tumour weight of the xenografts in each group. **(E)** Growth curves of the subcutaneous xenografts in each group. **(F)** The expression levels of METTL3, PAX8, NIS and Ki67 in xenografts of each group were assessed by immunohistochemical staining. The scale bar is 100 μm. On the right side of the IHC photo is a chart of the IHC score of METTL3 and PAX8. Data represents mean ± SEM, *$p < 0.05$, **$p < 0.01$, ***$p < 0.001$, ****$p < 0.0001$.

Supplementary Table 1:

Variables	Low expression	High expression	χ^2	P
Age				
＜55	43	13	1.036	0.309
＞=55	41	19		
Sex				
Female	62	17	4.564	0.033
Male	22	15		
Multifocality				
Presrent	38	9	2.816	0.093
Absent	46	23		
Tumor stage				
$T_{1/2}$	40	27	12.831	＜0.001
$T_{3/4}$	44	5		
Lymph node metastasis				
N_0	32	22	8.752	＜0.001
$N1_{a/b}$	52	10		
AJCC stage				
I+II	62	31	7.755	0.005
III+IV	22	1		
Extrathyroidal extension				
Absent	38	23	6.594	0.010
Present	46	9		

Supplementary Table 2: qRT-PCR primer sequences in this study

Primer names	Primer sequence
β -actin-F	GCATGGGTCAGAAGGATTCCT
β -actin-R	TCGTCCCAGTTGGTGACGAT
METTL3-F	CAGTGCTACAGGATGACGGCTT
METTL3-R	CCGTCCTAATGATGCGCTGCAG
SLC5A5-F	CTCTGCTGGTGCTGGACATCTT
SLC5A5-R	GAGGTCTTCTACAGTGACTGCAG
PAX8-F	TCAACCTCCCTATGGACAGCTG
PAX8-R	GAGCCCATTGATGGAGTAGGTG
NKX2.1-F	CAGGACACCATGAGGAACAGCG
NKX2.1-R	GCCATGTTCTTGCTCACGTCCC
TG-F	CCAGTGGCTTCTCTTCCTGACT
TG-R	CCTTGGAGGAAGCGGATGGTTT
TPO-F	CACCAGGCTTTCTTCAGCCCAT
TPO-R	GGACAGCACAAAGAGCCTTTCC
HHEX-F	CCAGGTGAGATTCTCCAACGAC
HHEX-R	CTCCATTTAGCGCGTCGATTCTG
YTHDC1-F	TCAGGAGTTCGCCGAGATGTGT
YTHDC1-R	AGGATGGTGTGGAGGTTGTTCC
YTHDC2-F	GAAAGCTCCTGAACCTCCACCA
YTHDC2-R	GGTTCTACTGGCAAGTCAGCCA

Supplementary Table 3: shRNA sequences in this study

Primer names	Primer sequence
shMETTL3#1-F	CCGGGCCAAGGAACAATCCATTGTTCTCGAGAACAATGGATTGTTCCTTGGCTTTTTG
shMETTL3#1-R	AATTCAAAAAGCCAAGGAACAATCCATTGTTCTCGAGAACAATGGATTGTTCCTTGGC
shMETTL3#3-F	CCGGGCTGCACTTCAGACGAATTATCTCGAGATAATTCGTCTGAAGTGCAGCTTTTTG
shMETTL3#3-R	AATTCAAAAAGCTGCACTTCAGACGAATTATCTCGAGATAATTCGTCTGAAGTGCAGC

Supplementary Table 4: siRNA sequences in this study

Primer names	Primer sequence
si YTHDC1#1	GAAGUGGAUAGACGUGCAATT
si YTHDC1#2	GCAAGGAGUGUUAUCUUAATT
si YTHDC2#1	CCGUCUGUUUAGUAGACUUTT
si YTHDC2#2	GCCUUGGAUGUAAAUCUCUTT

Supplementary Table 5:pmirGlo dual-luciferase reporter

pmirGlo dual-luciferase reporter	sequence
PAX8 3'UTR WT pmirGLO	ttgccatggggacagtgggagcgactgagcaacaggaggact<br>cagcctgggacaggccccagagagtcacacaaaggaatcttta<br>tttattacatgaaaaataaccacaagtccagcattgcggcacact<br>ccctgtgtggttaatttaatgaaccatgaaagacaggatgaccttg<br>gacaaggccaaactgtcctccaagactccttaatgaggggcag<br>gagtcccagggaaagagaaccatgccatgctgaaaaagacaa<br>aattgaagaagaaatgtagcccccagccggtacccaccaaagg<br>agagaagaagcaatagccgaggaacttggggggatggcgaat<br>ggttcctgcccgggcccaaggggtgcacagggcacctccatg<br>gctccattattaacacaactctagcaattatggaccataagcactt<br>ccctccagcccacaagtcacagcctggtgccgaggctctcctca<br>ccagccacccagggagtcacct
PAX8 3'UTR mut1 pmirGLO	ttgccatggggTcagtgggagcgactgagcaacaggaggact<br>cagcctgggacaggccccagagagtcacacaaaggaatcttta<br>tttattacatgaaaaataaccacaagtccagcattgcggcacact<br>ccctgtgtggttaatttaatgaaccatgaaagacaggatgaccttg<br>gacaaggccaaactgtcctccaagactccttaatgaggggcag<br>gagtcccagggaaagagaaccatgccatgctgaaaaagacaa<br>aattgaagaagaaatgtagcccccagccggtacccaccaaagg<br>agagaagaagcaatagccgaggaacttggggggatggcgaat<br>ggttcctgcccgggcccaaggggtgcacagggcacctccatg<br>gctccattattaacacaactctagcaattatggaccataagcactt<br>ccctccagcccacaagtcacagcctggtgccgaggctctcctca<br>ccagccacccagggagtcacct
PAX8 3'UTR mut2 pmirGLO	ttgccatggggacagtgggagcgactgagcaacaggaggTct<br>cagcctgggacaggccccagagagtcacacaaaggaatcttta<br>tttattacatgaaaaataaccacaagtccagcattgcggcacact<br>ccctgtgtggttaatttaatgaaccatgaaagacaggatgaccttg<br>gacaaggccaaactgtcctccaagactccttaatgaggggcag<br>gagtcccagggaaagagaaccatgccatgctgaaaaagacaa<br>aattgaagaagaaatgtagcccccagccggtacccaccaaagg<br>agagaagaagcaatagccgaggaacttggggggatggcgaat<br>ggttcctgcccgggcccaaggggtgcacagggcacctccatg<br>gctccattattaacacaactctagcaattatggaccataagcactt<br>ccctccagcccacaagtcacagcctggtgccgaggctctcctca<br>ccagccacccagggagtcacct

**PAX8 3’UTR mut3 pmirGLO**	**ttgccatggggacagtgggagcgactgagcaacaggaggact<br>cagcctgggTcaggccccagagagtcacacaaaggaatcttta<br>tttattacatgaaaaataaccacaagtccagcattgcggcacact<br>ccctgtgtggttaatttaatgaaccatgaaagacaggatgaccttg<br>gacaaggccaaactgtcctccaagactccttaatgaggggcag<br>gagtcccagggaaagagaaccatgccatgctgaaaaagacaa<br>aattgaagaagaaatgtagcccccagccggtacccaccaaagg<br>agagaagaagcaatagccgaggaacttgggggatggcgaat<br>ggttcctgcccgggcccaaggggtgcacagggcacctccatg<br>gctccattattaacacaactctagcaattatggaccataagcactt<br>ccctccagcccacaagtcacagcctggtgccgaggctctcctca<br>ccagccacccagggagtcacct**
**PAX8 3’UTR mut4 pmirGLO**	**ttgccatggggTcagtgggagcgactgagcaacaggaggTct<br>cagcctgggTcaggccccagagagtcacacaaaggaatcttta<br>tttattacatgaaaaataaccacaagtccagcattgcggcacact<br>ccctgtgtggttaatttaatgaaccatgaaagacaggatgaccttg<br>gacaaggccaaactgtcctccaagactccttaatgaggggcag<br>gagtcccagggaaagagaaccatgccatgctgaaaaagacaa<br>aattgaagaagaaatgtagcccccagccggtacccaccaaagg<br>agagaagaagcaatagccgaggaacttgggggatggcgaat<br>ggttcctgcccgggcccaaggggtgcacagggcacctccatg<br>gctccattattaacacaactctagcaattatggaccataagcactt<br>ccctccagcccacaagtcacagcctggtgccgaggctctcctca<br>ccagccacccagggagtcacc**
